# Strategies aimed at preventing long-term opioid use in trauma and orthopaedic surgery: a scoping review

**DOI:** 10.1186/s12891-022-05044-y

**Published:** 2022-03-11

**Authors:** C. Côté, M. Bérubé, L. Moore, F. Lauzier, L. Tremblay, E. Belzile, M-O Martel, G. Pagé, Y. Beaulieu, A. M. Pinard, K. Perreault, C. Sirois, S. Grzelak, A. F. Turgeon

**Affiliations:** 1 Population Health and Optimal Health Practices Research Unit, Trauma – Emergency – Critical Care Medicine, Centre de Recherche du CHU de Québec – Université Laval (Hôpital de l’Enfant-Jésus), 1401 18e Rue, Québec City, Québec G1J 1Z4 Canada; 2 Faculty of Nursing, Université Laval, 1050 Avenue de la Médecine, Québec City, Québec G1V 0A6 Canada; 3 Department of Social and Preventive Medicine, Faculty of Medicine, Université Laval, 1050 Avenue de la Médecine, Québec City, Québec G1V 0A6 Canada; 4 Department of Anesthesiology and Critical Care Medicine, Université Laval, 1050 Avenue de la Médecine, Québec City, Québec G1V 0A6 Canada; 5 Division of General Surgery, Sunnybrook Health Sciences Centre, 2075 Bayview Ave., Toronto, Ontario M4N 3M5 Canada; 6 Department of Orthopaedic Surgery, Université Laval, 1050 Avenue de la Médecine, Québec City, Québec G1V 0A6 Canada; 7 Faculty of Dentistry & Department of Anesthesia, McGill University, 1010 Rue Sherbrooke Ouest, Montreal, Québec H3A 2R7 Canada; 8 Research Center of the Centre hospitalier de l’Université de Montréal (CRCHUM), 850 rue St-Denis, Montreal, Québec H2X 0A9 Canada; 9 Department of Anesthesiology and Pain Medicine, Faculty of Medicine, Université de Montréal, 2900 Edouard Montpetit Blvd, Montreal, Québec H3T 1J4 Canada; 10 Center for Interdisciplinary Research in Rehabilitation and Social Integration, Centre intégré universitaire de santé et de services sociaux de la Capitale-Nationale, 525, boul. Wilfrid-Hamel, Québec City, Québec G1M 2S8 Canada; 11 Department of Rehabilitation, Faculty of Medicine, Université Laval, 1050 Avenue de la Médecine, Québec City, Québec G1V 0A6 Canada; 12 Faculty of Pharmacy, Université Laval, 1050 Avenue de la Médecine, Québec City, Québec G1V 0A6 Canada

**Keywords:** Opioids, Preventive strategies, Trauma, Orthopaedic surgery

## Abstract

**Background:**

Long-term opioid use, which may have significant individual and societal impacts, has been documented in up to 20% of patients after trauma or orthopaedic surgery. The objectives of this scoping review were to systematically map the research on strategies aiming to prevent chronic opioid use in these populations and to identify knowledge gaps in this area.

**Methods:**

This scoping review is reported according to the Preferred Reporting Items for Systematic Reviews and Meta-Analyses extension for Scoping Reviews (PRISMA-ScR) Checklist. We searched seven databases and websites of relevant organizations. Selected studies and guidelines were published between January 2008 and September 2021. Preventive strategies were categorized as: system-based, pharmacological, educational, multimodal, and others. We summarized findings using measures of central tendency and frequency along with *p*-values. We also reported the level of evidence and the strength of recommendations presented in clinical guidelines.

**Results:**

A total of 391 studies met the inclusion criteria after initial screening from which 66 studies and 20 guidelines were selected. Studies mainly focused on orthopaedic surgery (62,1%), trauma (30.3%) and spine surgery (7.6%). Among system-based strategies, hospital-based individualized opioid tapering protocols, and regulation initiatives limiting the prescription of opioids were associated with statistically significant decreases in morphine equivalent doses (MEDs) at 1 to 3 months following trauma and orthopaedic surgery. Among pharmacological strategies, only the use of non-steroidal anti-inflammatory drugs and beta blockers led to a significant reduction in MEDs up to 12 months after orthopaedic surgery. Most studies on educational strategies, multimodal strategies and psychological strategies were associated with significant reductions in MEDs beyond 1 month. The majority of recommendations from clinical practice guidelines were of low level of evidence.

**Conclusions:**

This scoping review advances knowledge on existing strategies to prevent long-term opioid use in trauma and orthopaedic surgery patients. We observed that system-based, educational, multimodal and psychological strategies are the most promising. Future research should focus on determining which strategies should be implemented particularly in trauma patients at high risk for long-term use, testing those that can promote a judicious prescription of opioids while preventing an illicit use, and evaluating their effects on relevant patient-reported and social outcomes.

**Supplementary Information:**

The online version contains supplementary material available at 10.1186/s12891-022-05044-y.

## Background

Considering the pain induced by traumatic injuries and surgery, opioids are often used in the early recovery phase of patients facing these health issues [1]. As such, a majority of trauma and surgical patients, particularly those who underwent orthopaedic procedures, still receive this analgesic at the time of hospital discharge and, alarmingly, up to 20% become chronic opioid users [2–6]. Moreover, the proportion can even reach 60% in those with a history of long-term opioid use [4, 7–9]. In this regard, several studies have documented risk factors for long-term opioid use in trauma and surgical patients, including prolonged duration of the initial opioid prescription [10–13], low income [9, 14], prior substance abuse [9, 14–18], use of specific medications (e.g., benzodiazepines, muscle relaxants, antidepressants) [9, 15, 18], psychologic comorbidities (particularly depression) [14, 15, 17–20], a history of chronic pain [15, 16, 18, 19, 21], and disease severity factors (e.g., complexity of fractures, invasiveness of spine surgery, number of surgeries, hospital length of stay) [17, 19, 20, 22].

The long-term use of opioids is associated with important individual and social negative consequences, which increase incrementally with the duration of opioid prescription [23]. For example, patients using opioids in the context of persistent pain were shown to be two to five times more likely to suffer from drowsiness, sleep disorders, headaches and constipation, compared to those not taking such medication [24]. Likewise, compared to nonopioid users, chronic users have greater psychological distress [2], greater interference with activities [2] and poorer quality of life [25, 26], without significantly improving their pain relief [2, 23, 25, 27]. Long-term opioid use is also associated with a 30% average rate of misuse (i.e., using opioids differently from how they are prescribed regardless of the presence of adverse events) [28]. Even more disturbing, long-term prescription may ultimately lead patients to the illicit purchase of opioids or its derivatives (e.g., heroin) to meet the needs of their addiction or to compensate for a decrease in prescribed doses [29–31]. Taken together, these issues were acknowledged to contribute to the increasing number of overdoses and deaths associated with opioids [32, 33].

Hence, strategies that promote a judicious use of opioids while still providing pain relief are needed during patients’ recovery phase to prevent subsequent long-term negative impacts. Strategies to decrease the amount of opioids used in patients already on chronic therapy, such as tools to improve opioid prescription, education for patients and health professionals, and interprofessional collaboration, have shown promising results [34]. However, little is known about available preventive strategies. Accordingly, we conducted a scoping review to systematically map the research done in this area, as well as to identify gaps in current knowledge on strategies to prevent chronic opioid use in adult trauma patients and in those who underwent an acute surgery for their injuries or a programmed orthopeadic surgery.

## Methods and analysis

Our scoping review was performed according to recommendations [35–37] and is reported according to the Preferred Reporting Items for Systematic Reviews and Meta-Analyses extension for Scoping Reviews (Supplemental Digital File 1: Preferred Reporting Items for Systematic reviews and Meta-Analyses extension for Scoping Reviews (PRISMA-ScR) Checklist) [38]. The study protocol was recently published [39].

### Eligibility criteria

We included randomized controlled trials (RCTs), quasi-randomized, prospective and retrospective observational cohorts, cross-sectional, case–control studies and guidelines. Preventive strategies (pharmacological or non-pharmacological) initially needed to target the acute care trajectory (from hospital admission to 3 months postinjury or post-surgery) [40, 41] of adult patients (≥ 18 years old) after traumatic injuries or acute care surgery [42]. We also included studies of elective orthopedic surgery patients, considering that the mechanisms of pain and likelihood of secondary opioid use are comparable to those of trauma patients that often have fractures [43]. Comparators included placebo, any other intervention, or standard treatment. We first considered outcomes related to opioid use measured at ≥3 months after trauma or surgery, as this timeframe is indicative of a chronic use or long-term therapy [19, 20]. Nonetheless, some studies measured outcomes at 1 month and 6 weeks (medium-term therapy) [44], prompting us to report results at these time points as well. Since opioids are administered for pain management, we also included outcomes related to pain intensity. We did not apply any language restriction.

### Data sources

Concerns with regard to adverse events associated with prescribed opioids and initiatives to limit long-term opioid use [40, 45] started shortly before 2010. Therefore, we systematically searched studies published between January 2005 and September 2021 registered in the following databases: MEDLINE, EMBASE, PsycINFO, CINAHL, the Cochrane Central Register of Controlled Trials (CENTRAL), Web of Science and ProQuest. As described in the published protocol [39], we also queried trauma and surgery, pain, government, and the websites of professional organisations. The reference lists of included articles were screened for any further eligible studies. Using Cochrane guidelines [46], we developed a rigorous systematic search strategy in collaboration with an information specialist. We used combinations of search terms under the themes of opioids and preventive strategies, including text terms and MESH (Medline) or EMTREE (Embase). We then adapted our search strategies for the other databases. The complete Medline search strategy is presented in Supplemental Digital File 2: Search Strategy in Medline.

### Selection and data charting processes

All citations were managed in Covidence (Veritas Health Innovation, Melbourne, Australia). After a reliability test on two sets of 100 citations, pairs of reviewers (MB, CC, SG, OS) independently screened all identified citations using titles, abstracts and full texts. Disagreements were settled through discussion between reviewers and further discussed with an expert clinical researcher when needed (AT). Two data extraction forms were created: one for original studies and one for practice guidelines. They were tested on a sample of five studies and two guidelines, respectively, before pairs of reviewers (MB, CC, SG and OS) independently extracted data. The following information was retrieved from original studies: setting, population, risk factors for chronic opioid use (e.g. history of substance abuse, chronic pain, mood disorders) [9], study design, intervention(s), comparator(s), outcome measures and their timepoints, effectiveness of the strategy based on outcome measures of central tendency (i.e., mean) and frequency (rate, proportion) in intervention and comparator groups along with *p*-values for statistical significance. The same pairs of reviewers also extracted the following data from guidelines: setting, population and the recommended preventive strategies along with their level of evidence and the strength of their recommendations.

### Data items

Data collation was conducted independently by two reviewers (CC and SG) and validated by a third reviewer (MB). Preventive strategies were organized into seven categories according to the type of preventive strategy as per pain management guidelines in trauma [47] and other fields [48, 49]: system-based, pharmacological, educational, multimodal and others, which included surgical procedures, alternative and psychological. The evidence from original studies was categorized based on whether or not findings favoured preventive strategies, as demonstrated by statistically significant results, set at *p* < 0.05 in the context of this review. We also compared the outcomes for the populations included in this scoping review (trauma, spine surgery, and elective orthopedic surgery) in relation to the categories of the preventive strategies. Results for the trauma population were not presented separately because of the similarities in the data compared to other populations and the fact that some categories or specific types of intervention contained very few studies. For guidelines, we described recommendations according to preventive strategy categories and their level of evidence specifying to which populations they applied. We reported levels of evidence and strength of recommendations according to the classification systems used in each of the guidelines.

## Results

### Literature search and selection process

**Fig. 1:**
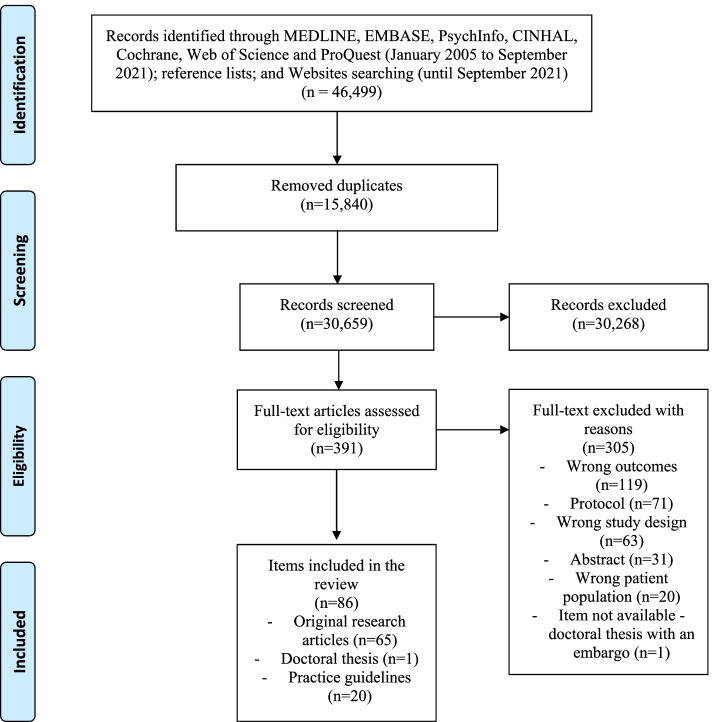
Flow diagram on evidence screened, assessed for eligibility, and included in the review

The searches identified 46,499 citations, including 15,840 duplicates of original studies and guidelines. As shown in Fig. 1, 391 studies met the inclusion criteria after initial screening. A total of 308 studies were excluded after the full-text review and are listed in Supplemental Digital File 3: Excluded full texts. The main reasons for exclusion at the full-text stage were: not measuring opioid use (*n* = 114), having only protocols available (*n* = 71) or wrong study design (*n* = 66). After final screening we included 66 studies [5, 50–114] and 20 [47, 48, 115–132] guidelines in the qualitative synthesis. These studies and guidelines were published between 2008 and 2021, with only one item (1.2%) published before 2010, 16 (18.6%) between 2010 and 2016 and 69 (80.2%) between 2017 and 2021.

### Study and guideline characteristics

The key characteristics of the 66 included studies [5, 50–114] are detailed in Table 1: Study Characteristics, Description of Strategies and Outcomes. Most studies used a retrospective cohort (*n* = 35) [5, 52, 56, 60, 72–74, 78–89, 91–94, 97–99, 101–105, 108, 109, 111, 112], a randomized controlled trial (RCT) (*n* = 24) [50, 51, 53–55, 57, 59, 61, 62, 65–71, 75, 76, 95, 100, 106, 107, 113, 114] or a prospective cohort (*n* = 7) [58, 63, 64, 77, 90, 96, 110] design. Almost all the studies were conducted in the U.S. (*n* = 58) [5, 50–57, 59–61, 63, 67–74, 77–99, 101–114].

**Table 1:** Study characteristics, description of strategies and outcomes

First author, year, country	Design	Sample size	Age in years mean	Female%	Type of trauma or surgical procedure	Intervention (strategies)	Comparator	Outcomes	Results Mean or median, % (statistically significant results favoring intervention are in bold)^a^
**Studies including trauma patients**									
**System-based**									
Chambers 2021 USA [96]	Prospective cohort	86	37	40%	Outpatient orthopaedic trauma surgery	Implementation of the Orthopaedic Trauma Association (OTA) pain management guidelines for acute musculoskeletal injuries	Before guidelines implementation	Cumulative MED	*6 weeks:* I; 210.00; C: 225.00, 95% CI -85.00 - 20.00, *p* = 0.10
								Proportion of patients who received opioid refill(s)	I: 2.00%; C: 2.00%, 95% CI -9.00 - 8.00, *p* = 1.00
								Proportion of patients using opioids (adherent vs non-adherent to guidelines for discharge prescription)	*6 weeks:* Adherent: 17.00%; Non-adherent: 13.00% 95% CI − 22.00 - 13.00 *p* = 0.70
Chen 2020 USA [77]	Prospective cohort	2,940	57	53%	Orthopaedic surgery including for traumatic fractures	A patient-specific protocol using an opioid taper calculator to standardize opioid prescribing at discharge after inpatient orthopaedic surgery	Before protocol implementation	MED	*At discharge:* I: **326.00**; C: 427.00, *p* < 0.001
								Refills mean	*1 month:* I: 1.71; C: 1.58, *p* = 0.08
Reid 2020 USA [87]	Retrospective cohort	753	57	56%	Orthopaedic trauma	State of Rhode Island legislation on strict opioid prescription limits. These limits prohibited providers from prescribing more than 30 MED per day, 150 total MED, or 20 total doses initially following a surgical procedure.	Idem as Reid 2019	Cumulative MED	*1 month:* I: **481.70**; C: 677.40, *p* < 0.001 Opioid-tolerant: I: **880.00**; C: 1,659.20, *p* = 0.04 Opioid-naïve: I: **478.10**; C: 633.70, *p* < 0.001 *30-90 days*: I: 265.10; C: 256.70, *p* = 0.83 Opioid-tolerant: I: 923.80; C: 1,691.10, *p* = 0.10 Opioid-naïve: I: 241.10; C: 206.90, *p* = 0.90
Wyles 2020 USA [97]	Retrospective	4,523	63	51%	Orthopaedic and spine surgery including for traumatic fractures	Implementation of procedure-specific guidelines for discharge opioid prescriptions	Before guidelines implementation	MED	*AT discharge:* I: **375.00**; C: 600.00 *p* < 0.001
								Proportion of patients who received opioid refill(s)	*1 month:* I: 24.00%; C: 25.00% *p* = 0.43
Choo 2019 USA [79]	Retrospective cohort	830	63	48%	Orthopaedic surgery including for traumatic fractures	A quality improvement project using report sent to health professionals every two months, which showed median discharge MED per patient and reinforcement on multimodal pain management strategies	Before quality improvement project implementation	MED	*At discharge:* I: **450.00**; C: 600.00, *p* < 0.001
								Proportion of patients who received opioid refill(s)	*Between discharge to 1 month*: I: 24.00%; C: 25.70%, *p* = 0.58 *Between 1 to 2 months*: I: 14.90%; C: 14.20%, *p* = 0.77 *Between 2 to 3 months*: I: 7.80%; C: 6.50%, *p* = 0.58
Reid 2019 USA [86]	Retrospective cohort	1,776	55	55%	Orthopaedic surgery including for traumatic fractures	Idem as Reid 2019	Before legislation implementation	Cumulative MED	*1 month*: I: **524.50**; C: 790.00, *p* < 0.001 Opioid-Tolerant: I: **1,015.20**; C: 1,304.10, *p* = 0.001 Opioid-Naïve: I: **446.57**; C: 708.84, *p* < 0.001 *Between 1 to 3 months*: I: **208.50**; C: 243.50, *p* = 0.007 Opioid-Tolerant: I: 804.60; C: 892.60, *p* = 0.08 Opioid-Naïve: I: **113.70**; C: 141.00, *p* = 0.02
								Proportion of patients using opioids	*At 30 days:* I: **24.00%**; C: 28.00%, *p* = 0.03
Young 2019 USA [93]	Retrospective cohort	218	75	72%	Minor non-surgical trauma	After Ohio's opioid prescription limit (opioids for 7 days and a total of 210 MEDs)	Before opioid prescription limit	Cumulative MED	*1 month:* I: **105.00**; C: 375.00, *p* = 0.02
Earp 2018 USA [80]	Retrospective cohort	518	54	61%	Hand and upper-extremity surgeries including for traumatic fractures	Postoperative opioid-limit prescribing protocol	Before protocol implementation	MED	*At discharge* Tier^b^ 1: I: **39.20**; C: 113.60, *p* < 0.001 Tier 2: I: **61.40**; C: 171.10, *p* < 0.001 Tier 3: I: **131.20**; C: 229.60, *p* < 0.001 Tier 4: I: **208.10;** C: 264.80, *p* < 0.02 Tier 5: I: **246.90;** C: 369.90, *p* < 0.003 MED decreased by a minimum of 97.80% and a maximum of 176.00% (*p* < 0.05 for all tiers)
								Proportion of patients who received opioid refill	1 refill: I: **1.70%**; C: 6.50%, *p* < 0.001 2 refills: I: **0.00%**; C: 1.70%, *p* < 0.001
**Pharmacological**									
Cunningham 2021 USA [98]	Retrospective cohort	230	64	65%	Distal femur fracture surgery	Regional anesthesia	Without regional anesthesia	Cumulative MED	*6 weeks:* I: 95.10; C:**74.90** Incident rate ratio : **1.27**, 95% CI 1.01-1.59, *p* = 0.03 *3 months:* I: 112.10; C:**85.00** Incident rate ratio : **1.33**, 95% CI (1.07, 1.66), *p* = 0.01 *Between 6 weeks to 3 months*: I : OR **1.85** 95% IC (1.14, 3.04) *p* = 0.014
Cunningham 2021 USA [99]	Retrospective	230	41	35%	Pelvis and acetabulum fracture surgery	Regional anesthesia	Without regional anesthesia	Cumulative MED	*6 weeks:* I: 177.20; C:145.20 Incident rate ratio : 1.22, 95% CI 0.99-1.51, *p* = 0.06 *3-months:* I: 207.90; C:**156.80** Incident rate ratio **1.33**, 95% CI 1.06-1.65, *p* = 0.01
								Opioid fill	Between 6 weeks to 3 months: I : OR **2.05**, 95% CI 1.24- 3.46, *p* = 0.006
Bhashyam 2018 USA [63]	Prospective cohort	500	50	50%	Orthopaedic trauma	Recreational use or self-medication with marijuana I1: Prior user I2: Use during recovery	Never use marijuana	Total prescribed MED	*6 months*: Marijuana used during recovery compared to never users (mean difference = **343.00**, *p* = 0.03)
								Duration of opioid use (days)	Marijuana used during recovery compared to never users (mean difference = **12.50**, *p* = 0.03)
								Proportion of patients using opioids (%)	*Persistent Use for > 3 months :* I1: 25.90%; I2: 21.70%; C: 17.60%, no significance test
Radi 2017 USA [94]	Retrospective cohort	216	NS	37%	Orthopaedic trauma	Peri-operative regional nerve block (single shot)	No block	Proportion of patients using opioids	*3 months*: I: 44.20%; C: 34.80%, *p* = 0.22 *6 months*: I: 7.70%; C: 14.60%, *p* = 0.19
Yazdani 2016 Iran [75]	Randomized controlled trial	60	32	17%	Trauma: ORIF of a recent mandibular unilateral body fracture	A 100 mg dose of Amantadine one hour before surgery	Placebo capsule	Cumulative MED	*6 months*: I: 121.70; C: 106.00, *p* = 0.61
Gray 2011 Australia [66]	Randomized controlled trial	90	36	17%	Burn injury	Pregabalin (75 mg to 300 mg titration according to pain level) twice daily for 28 days and weaned and ceased over the next 6 days.	Placebo capsules	Morphine Parenteral Equivalent/ day	*1 month:* I: 14.92; C: 14.92, *p* = 0.09
**Educational**									
Bérubé 2021 Canada [100]	Randomized controlled trial	49	41	25%	Traumatic injury requiring hospital- ization. Patients receiving > 2 doses/ day of opioid at discharge and with at least one risk factor for chronic opioid use	TOPP-Trauma programme + UC. This educational program (2 x 10 min session prior discharge and max 6 x 15 min opioid tapering counselling session every 2 weeks after discharge) focused on multimodal pain management strategies and guidance about opioid tapering	UC + an educational pamphlet received before discharge	Reported MED/day	*6 weeks:* I: 1.20; C: 12.20, 95% CI –22.00-0.10 *3 months :* I: 0.40; C: 4.10, 95% CI – 8.30-0.70
								Total MED delivered	*6 weeks:* I: 618.19; C: 1,009.00, 95% CI –1,324.00-542.10 *3 months :* I: 679.00; C: 1,443.40, 95% CI – 1,781.60-248.60
								Proportion of patients using opioids (%)	*6 weeks:* I: 17.00%; C: 29.00%, *p* > 0.05 3 months: I: 12.00%; C: 16.00%, *p* > 0.05
Syed 2018 USA [53]	Randomized controlled trial	134	59	32%	Arthroscopic rotator cuff repair	Formal education detailing recommended postoperative opioid usage, side effects, dependence, and addiction	Preoperative education regarding surgery	Cumulative MED	*6 weeks*: I: **40.40**; C: 60.60, *p* = 0.02 *3 months*: I: **51.20**; C: 87.20, *p* = 0.01
								Opioids discontinua-tion	*Between 6 weeks to 3 months*: OR: **2.19**, 95% CI 1.10-4.39, *p* = 0.03
Stanek 2015 USA [52]	Retrospective cohort	NS	NS	NS	Hand surgery including for traumatic fractures	Implementation of an educational assist device to serve as a memory prompt of narcotic guidelines	Before implementation of the educational assist device	Reduction in opioid prescription (%)	*3 months*: Repair of a metacarpal fracture: **20.00%** reduction, *p* = 0.04
Holman 2014 USA [5]	Retrospective cohort	613	43	38%	Orthopaedic trauma	A standardized discussion with patients aiming to inform them that they would receive opioids for a maximum of 6 weeks postoperatively	No standardized discussion but limited postoperative opioids prescriptions to 12 weeks	Proportion of patients using opioids	*6 weeks:* I: **27.00%**; C: 36.00%, *p* = 0.01 *3 months*: I: 20.00%; C: 20.00%, *p* = 0.90
**Multimodal**									
Singer 2021 USA [101]	Retrospective cohort	620	49	32%	Hospitalized trauma patients	Multimodal analgesia protocol and corresponding electronic medical record order set (including opioids, NSAID and gabapentin who were adjusted for age and medical condition)	Before implementation of multimodal protocol.	Cumulative outpatient MED	*6 months:* I: **210.00**; C: 263.00, *p* = 0.03
								Proportion of patients using opioids chronically (opioid prescription at 6 mo)	I: 3.20%; C:3.10%, *p* = 0.62
**Alternatives**									
Crawford 2019 USA [55]	Randomized controlled trial	233	45	39%	Lower extremity surgery including for traumatic injuries (military population)	Standard care and modified battlefield acupuncture with semi-permanent needles	C1: standard care + small adhesive bandages on the ear C2**:** standard care + placebo auricular acupuncture with semi-permanent needles	Cumulative MED	*1 month*: I: 257.00; C1: 358.00; C2: 266.00, *p* = 0.22
**Psychological**									
**Studies including non-trauma surgical patients: orthopeadic and spine**									
**System-based**									
Chalmers 2021 USA [102]	Retrospective cohort	19428	63	53%	THA or TKA	Modification of routine discharge MED (C = 750 MED, I1 = 520 MED, I2 = 320 MED)	Before routine discharge reduction (C)	Cumulative MED (mean)	*3 months* Total population: I1: **798.00**; I2: **556.00**; *C:* 1,009.00, *p* < 0.001
								Postoperative refill in MED (mean)	Total population: I1: **859.00**; I2: **682.00**; *C:* 1,017.00, *p* < 0.001
								Proportion of patients who received opioid refill(s)	Total population: I1: **33.00%;** I2: **33.00%;** C: 28.00%, *p* < 0.001
Cunningham 2021 USA [103]	Retrospective cohort	4,592	61	57%	ACDF, ACLR, CTR, RCR, TAA, THA, TKA, trapeziec-tomy with suspension-plasty	North Carolina legislation. The STOP Act requires to review a patient’s 12-month history before issuing an initial prescription for an opioid and instituting a 5-day limit on initial prescriptions for acute pain and a 7-day limit on postoperative prescriptions + institutional educational materials for practitioners and patients about responsible opioid prescribing, opioid use, and North Carolina law (I1: immediately after implementation; I2: 1 year after implementation)	Before implementation of the STOP Act legislation and departmental policies (C)	Total MED prescribed	*6 weeks:* I1:**126.15**; I2: **120.30**; C: 184.95, *p* < 0.001
								Proportion of patients who received more than one prescription	I1: **30.50%;** I2: **31.70%;** C: 37.20%, *p* < 0.001
Raji 2021 USA [104]	Retrospective case-control	334	69	65%	Different types of shoulder arthroplasty	After Ohio legislation which limit opioid prescriptions to no more than 7 days at a time for adults, with a maximum allotted dose per day of 30 morphine milligram equivalents	Before implementation of Ohio legislation	Total MED	*1 month:* Total: I: **300.00**; C: 570.00, *p* < 0.001 Opioid tolerant: I: 740.00; C: 825.00, *p* = 0.551 Oioid naïve: I: **210.00**; C: 450.00, *p* < 0.001 *Between 1 to 2 months:* Total: I: 0.00; C: 0.00, *p* = 0.88 Opioid tolerant: I: 360.00; C: 300.00, *p* = 0.449 Oioid naïve: I: 0.00; C: 0.00, *p* = 0.779 *Between 2 to 3 months:* Total: I: 0.00; C: 0.00, *p* = 0.47 Opioid tolerant: I: 405.00; C: 300.00, *p* = 0.506 Oioid naïve: I: 0.00; C: 0.00, *p* = 0.853 *Between discharge to 3 months:* Total: I: **450.00**; C: 600.00, *p* < 0.001 Opioid tolerant: I: 1,680.00; C: 1,455.00, *p* = 0.802 Oioid naïve: I: **210.00**; C: 487.50, *p* < 0.001
Sabesan 2021 USA [105]	Retrospective cohort	143	73	56%	Primary reverse shoulder arthroplasty	After Florida House bill 21 law (restriction of 3 to 7-days supply of opiates for acute pain)	Before House Bill 21 law.	Cumulative MED	*3 months:* I: **461.90**; 1750.7, *p* = 0.035
								Proportion of patients who received opioid refill(s)	I: **17.80**%; C: 70.1%, *p* < 0.001
								Proportion of patients using opioids chronically (for 3 or more months of continuous usage)	I: **12.50**%; C: 23.00%, *p* < 0.043
Eley 2020 USA [81]	Retrospective cohort	246	59	38%	Spine surgery	Implementation of an opioid prescription-limit protocol	Before protocol implementation	MED	*At discharge:* I: **120.60**; C: 286.90, *p* < 0.001
								Proportion of patients who received opioid refill	*3 months:* I: 17.10%; C: 16.50%, *p* = 0.98
								Proportion of patients transitioning to chronic opioid use	I: 2.40%; C: 4.60%, *p* = 0.70
Joo 2020 USA [83]	Retrospective cohort	83	67	1%	Spine surgery	An individualized discharge opioid prescribing and tapering protocol	Before protocol implementation	Cumulative MED (median) Proportion of patients who received opioid refill(s)	*6 months*: I:**300.00**; C:900.00, *p* < 0.01 I: 36.80%; C: 40.00%, *p* = 0.77
Tamboli 2020 USA [89]	Retrospective cohort	49	68	8%	THA	Multidisciplinary patient-specific opioid prescribing and tapering protocol	Before protocol implementation	Cumulative MED (median)	*6 weeks*: I: **295.00**; C: 900.00 MD: 721, 95% CI 127.00-1,316.00, *p* = 0.007
								Proportion of patients who received opioid refill	I: 54.00%; C: 48.00%, *p* = 0.67 > 1 refill: I: 54.00%; C: 67.00%, *p* = 0.69
Whale 2020 USA [91]	Retrospective cohort	1,994	68	62%	THA or TKA	After Ohio Opioid Prescribing Guidelines	Before implementation of prescribing guidelines	Cumulative MED	Total (acute and chronic follow-ups): TKA cohort: All: I: **1,145.80**; C: 1,602.60, *p* < 0.01 THA cohort: All: I: **878.30**; C: 1,302.30, *p* < 0.01 *Between discharge to < 3 months (acute)* TKA cohort: I: **390.70**; C: 519.70, *p* = 0.02 THA cohort: I: 178.60; C: 232.10, *p* = 0.27 *≥ 3 month (chronic)* TKA cohort: All: I: 148.80; C: 178.10, *p* = 0.48 THA cohort: All: I: 69.00; C: 121.80, *p* = 0 .12
								Proportion of patients who received opioid refill(s)	*Acute:* TKA: I: 47.20%; C: 41.50%, *p* = 0.50 THA: I: 25.70%; C: **18.30%**, *p* = 0.01 *Chronic:* TKA: I: 12.00%; C: 12.70%, *p* = 0.72 THA: I: 9.50%; C: 10.00%, *p* = 0.83
Chen 2019 USA [78]	Retrospective cohort	60,056	65	7%	TKA (veteran population)	Opioid safety initiative that combined education, guideline dissemination with audit and feedback using dashboards	Before opioid safety initiative implementation	Proportion of patients using opioids chronically (for greater than 3 months in a 6-month period)	*6 months:* Post-operative chronic user: I: **14.10%**; C: 26.90%, *p* < 0.001
Holte 2019 USA [82]	Retrospective cohort	399	61	52%	TKA and THA	Implementation of strict postoperative opioid prescription guidelines and mandatory preoperative patient education session led by nursing staff regarding postoperative pain management with an emphasis on opioid use	Before implementation of guidelines	MED	*At discharge:* I: **387.30**; C: 751.50 *p* < 0.0001
								Total postoperative refill in MED	*3 months:* I: **84.00**; C: 253.00, *p* = 0.004
								Number of refills (mean)	I: **0.30**; C: 0.50, *p* = 0.02
								Number of call-ins pertaining to pain management (mean)	I: **0.40**; C: 0.70, *p* = 0.03
Reid 2019 USA [88]	Retrospective cohort	1,125	67	62%	THA or TKA	State of Rhode Island legislation on strict opioid prescription limits. These limits prohibited providers from prescribing more than 30 MED per day, 150 total MED, or 20 total doses initially following a surgical procedure.	Before legislation implementation	Cumulative MED	*1 month*: I: **632.00**; C: 907.00, *p* < 0.001 Opioid-Tolerant: I: 1,288.00; C: 1,398.00, *p* = 0.06 Opioid-Naïve: I: **501.00**; C: 796.00, *p* < 0.001 *1to 3 months*: I: 270.00; C: 279.00, *p* = 0.19 Opioid-Tolerant: I: 1,119.00; C: 898.00, *p* = 0.96 Opioid-Naïve: I: 100.00; C: 139.00, *p* = 0.17
								Number of refills	*1 month*: I: 2.20; C:**1.90,** *p* < 0.001 Opioid-Tolerant: I: **3.0**; C: 2.50, *p* = 0.03 Opioid-Naïve: I: 2.10; C: **1.80,** *p* < 0.001
Reid 2019 USA [85]	Retrospective cohort	211	52	54%	Spine Surgery	Idem	Idem	Number of prescriptions (n)	*1 month:* I: 1.70; C:1.60, *p* = 0.42
								Cumulative MED	*1 month*: I: **444.10**; C: 877.90, *p* < 0.001 Opioid-Tolerant: I: **632.20**; C: 1,122.90, *p* < 0.001 Opioid-Naïve: I: **363.40**; C: 730.10, *p* < 0.001 *Between 1 to 2 months:* I: 129.50; C: 181.00, *p* = 0.25 Opioid-Tolerant: I: 407.90; C: 546.20, *p* = 0.23 Opioid-Naïve: I: 150.30; C: 207.00, *p* = 0.13 *61 to 90 days*: I: 91.90; C: 153.60, *p* = 0.19 Opioid-Tolerant: I: 226.70; C: 272.20, *p* = 0.82 Opioid-Naïve: I: 87.90; C: 126.10, *p* = 0.30 *91 to 120 days*: I: 131.20; C: 136.80, *p* = 0.08 Opioid-Tolerant: I: 181.20; C: 274.00, *p* = 0.21 Opioid-Naïve: I: 53.70; C: 81.00, *p* = 0.07
Vaz 2019 USA [90]	Prospective cohort	196	68	58%	THA or TKA	Standardized opioid prescription protocol: maximum of 30 pills (370 MED) for THA and 40 pills (490 MED) for TKA	Postoperative analgesic prescription at provider’s discretion	Cumulative MED	*1 month*: TKA cohort: I: **200.00**; C: 504.00, *p* < 0.001 THA cohort: I: **432.00**; C: 902.00, *p* < 0.001
								Proportion of patients who received opioid refill(s)	TKA cohort: I: 50.00%; C: **29.00%**, *p* = 0.04 THA Cohort: I: 16.00%; C: 8.00%, *p* = 0.2
Wyles 2019 USA [92]	Retrospective cohort	2573	67	53%	TKA or THA	Clinicians were recommended to prescribe a maximum MED for an opioid prescription based on the procedure level: Level 1 = 100 MED, Level 2 = 200 MED, Level 3 = 300 MED, and Level 4= 400 MED	Prescriptions without guidelines	Cumulative MED (median)	*1 month* (median): TKA cohort: I: **388.00**; C: 750.00, *p* < 0.001 THA cohort: I: **388.00**; C: 750.00, *p* < 0.001
								Proportion of patients who received opioid refill(s)	TKA cohort: I: 35.00%; C: 35.00%, *p* = 0.77 THA cohort: I: 17.00%; C: 16.00%, *p* = 0.55
**Pharmacological**									
Burns 2021 USA [106]	Randomized controlled trial	157	61	52%	Scheduled shoulder arthroplasty (group 1) or ARCR (group 2)	Celecoxib 200 mg twice daily for 3 weeks	Placebo medication	Difference in MED between I and C group (ß)	*6 weeks:* Total population: –**198.80** *p* = 0.01 Group 1 –**270.00** *p* = 0.04 Group 2: –94.50 *p* = 0.31
Zhuang 2020 China [76]	Randomized controlled trial	246	68	80%	TKA	Supplied sequential treatment with intravenous parecoxib 40 mg (every 12 hours) for the first 3 days after surgery, followed by oral celecoxib 200 mg (every 12 hours) for up to 6 weeks	Placebo medication	Cumulative MED (median)	*1 month*: I: **53.33**; C: 166.50 Median difference: **112.02**, 95% CI 43.12-150.92, *p* < 0.001 *6 months:* I: **58.00**; C: 180.35 Median difference: **120.92**, 95% CI 57.34-181.81, *p* < 0.001
Starr 2019 USA [72]	Randomized controlled trial	11,614	66	6%	TKA (veteran population)	β-blocker within 90 days prior to surgery, β-blocker as an inpatient on postoperative day 0 or 1, and refill prescription for a β-blocker within 90 days after surgery	No β-blocker	Cumulative MED	*1 month*: I: **86.10**; C: 90.40, *p* = 0.004
								Proportion of patients using opioids	*1 month:* OR **0.89**, 95% CI 0.80-0.99, *p* = 0.02 *3 months*: OR 1.00, 95% CI 0.87-1.15, *p* = 0.965 *12 months:* OR 1.04, 95% CI 0.90-1.20, *p* = 0.54
Fenten 2018 Netherlands [65]	Randomized controlled trial	153	65	54%	TKA	LIA of the posterior capsule and a FNB catheter	Periarticular LIA with ropivacaine 0.2% for postoperative analgesia	Proportion of patients using opioids	*3 months*: I: 7.90%; C: 13.00% No significance test *12 months*: I: 5.40%; C: 2.60% No significance test
Hah 2018 USA [67]	Randomized controlled trial	410	57	58%	Surgeries: orthopeadic (80% of patients), thoracotomy, and breast	Four capsules of gabapentin, 300mg preoperatively and two capsules of gabapentin, 300 mg, 3 times a day postoperatively (10 total doses)	Placebo capsules	Proportion of patients using opioids	*6 months:* I: 2.40%; C: 2.00% OR 1.22, 95% CI 0.32-4.66, *p* = 0.80 12 months: I: 1.90%; C: 1.50% OR 1.28, 95% CI 0.28-5.87, *p* = 0.70
Thompson 2018 USA [74]	Retrospective cohort	44	70	68%	TEA	Liposomal bupivacaine mixture through indwelling interscalene catheter	Indwelling interscalene catheter	Cumulative MED	*3 months:* I: 1,198.60; C: 1,762.50, *p* = 0.19
Sun 2017 USA [73]	Retrospective cohort	120,080	57	61%	TKA	Nerve Block	No nerve block	Proportion of patients using opioids chronically (having filled 10 or more prescriptions or >120 days’ supply within the first year of surgery, excluding the first 90 postoperative days)	*12 months:* Opioid naïve: I: 1.78%; C: 1.81%, *p* = 0.744 Adjusted for patient demographics, comorbidities, and preoperative medication use (ARR): 0.98, 98.3% CI 0.847-1.14, *p* = 0.79 Chronic user: I: 67.60%; C: 67.80%, *p* = 0.761 Intermittent user: I: 6.08%; C: 6.15%, *p* = 0.787
Hyer 2015 USA [69]	Randomized controlled trial	70	53	48%	Spinal surgery	Duloxetine once a day 2 weeks before and more then 3 months after surgery	Placebo capsule	Opioid use	*1 month:* *p* > 0.05
Aguirre 2012 Switzerland [62]	Randomized controlled trial	72	58	51%	Minimally invasive hip surgery	20 mL ropivacaine 0.3% applied into the wound as a bolus before wound closure followed with a continuous infusion of ropivacaine 0.3% at 8 mL/h for 48 hours after surgery	NaCl 0.9% placebo	Opioid use	*3 months*: *p* > 0.05
Nader 2012 USA [70]	Randomized controlled trial	62	65	70%	TKA	Continuous femoral analgesia for 24 hours	Oral opioid analgesia	Median daily MED	*1 month*: I: 10.00 mg; C: 18.00 mg, *p* = 0.12 *6 months*: I: 0.00; C: 0.00, *p* = 0.63
Chevet 2011 France [64]	Prospective cohort	107	72	72%	TKA	An intravenous dose of 15 mg/kg of ATX between induction and incision, renewed at the end of surgery	No ATX	Proportion of patients using mild opioids	*6 months*: I: 20.00%; C: 33.00%, *p* = 0.18
Schroer 2011 USA [71]	Randomized controlled trial	107	67	58%	TKA	Celecoxib 200 mg to twice daily for 6 weeks after discharge	Placebo capsules	Number of opioid pills used (dosage NS)	*12 months*: I: **76.30**; C: 138.00, *p* = 0.003
**Educational**									
Cheesman 2020 USA [107]	Randomized controlled trial	140	58	32%	ARCR	Formal opioid education (recommended postoperative opioid use, side effects, dependence, and addiction) + a 2-minute computer-based presentation concerning opioid abuse and its consequences + a paper outline on the most important points of the presentation	Standard preoperative education followed by a discussion of risks and benefits. No formal education on opioid use, dependence, and addiction.	Total MED	*24 months :* Total population: I: 375.00; C:725.00 *p* = 0.27 Opioid-naïve patients: I: 375.00; C: 535.00 *p* = 0.42 Prior opioid use: I: 1,612.00; C:2,475.00 *p* = 0.57
								Proportion of opioid dependence (6 opioid prescriptions from the date of surgery)	Total population: I: 11.40%; C: 25.70% *p* = 0.5 Opioid-naïve patients: I: **3.70**%; C: 16.70% *p* = 0.04 Prior opioid use: I: 37.50%; C: 47.60% *p* = 0.78
								No of prescriptions filled	Total population: I: **2.90**; C: 6.30 *p* = 0.03 Opioid-naïve patients: I: 1.20; C: 3.40 *p* = 0.6 Prior opioid use: I: 8.90; C: 13.20 *p* = 0.56
Campbell 2019 USA [50]	Randomized controlled trial	159	60	45%	THA or TKA	Traditional perioperative education + automated text messages included recovery instructions paired with encouraging and empathetic statements, personalized video messages from their surgeon, and short instructional videos	Traditional perioperative education, which included a preoperative clinic appointment and perioperative instructions	Time to opioids cessation (days)	*6 weeks:* I: **22.50**; C: 32.40 Mean difference: -10.0, 95% CI -14.2-(-5.7), *p* < 0.001
Smith 2018 USA [51]	Randomized controlled trial	561	66	60%	TKA or THA	Usual care + pharmacist intervention Usual care: an educational session that advised patients on the risks and benefits of surgery, pain control measures and exercise recommendations. Pharmacist intervention: mailed brochures describing what patients should expect regarding opioid use and pain control after and follow-up telephone call from a pharmacist.	Usual Care (handouts and a class in preparation for surgery that advised patients on the risks and benefits of surgery, pain control measures, exercise recommenda-tions, and the need for postsurgical assistance)	Total dispensing of opioid medications	*3 months*: Adjusted mean difference for patients sociodemographics and probability of long-term opioid use: 0.92 95% CI 0.69-1.21 No readmission for pain control during the study period.
**Multimodal**									
Urban 2021 USA [108]	Retrospective cohort	267	67	63%	TKA	Preoperative cryoneurolysis (1 min 45 sec cycle in the infrapatellar branches of the saphenous nerve near the knee and branches of the femoral cutaneous nerves in the mid-to-distal anterior thigh + standard multimodal regiment.	Standard multimodal regiment (preoperative protocol + postoperative : oral acetaminophen 500 mg every 6 hours, oral meloxicam 7.5mg twice daily, oral tramadol 50 mg every 6 hours as needed for pain, oral oxycodone 5 mg every 3 hours as needed)	Cumulative MED	*6 weeks:* Mean: I: **894;** C: 1,406.00 Ratio estimate : **0.64** 95% CI 0.57-0.71, *p* < 0.001
								Proportion of patients who received ≥1 prescription at 6 weeks	I: 12.00%; C: 20.00% Ratio estimate : 0.61 95% CI 0.29-1.28, *p* < 0.19
Buys 2020 USA [109]	Retrospective cohorte	336	65	10 %	RCR, THA, TKA, TSA (veteran population)	Implementation of a Transitional Pain Service. Multidisciplinary providers work together to deliver comprehensive pain management for any surgical patient at risk for CPSP and COU in preoperative, surgical hospitalization and postoperative period up to 6 months.	Before Transitional Pain Service implementation	Proportion of patients still using opioids	*3 months:* Patients with history of COU I: **33.40%;** C: 23.30% *p* = 0.002 Opioid-naïve patients I: **0.70%;** C: 8.40% *p* = 0.004
Li 2020 USA [110]	Prospective cohort	143	66	45%	TKA	Multimodal pain management + opioid PRN	Opioid-only analgesia	Cumulative MED	*1 month:* *Mean:* I: **386.40**; C: 582.50 *p* = 0.0006
								Proportion of patients who required a refill	I: **51.40**%; C; 74.60% *p* = 0.004
Fleischman 2019 USA [57]	Randomized controlled trial	235	63	46%	THA	**I1:** Multimodal analgesic regiment (acetaminophen 1000 mg tid x 4w + Gabapentin 200 mg bid x 4 w + Meloxicam 15 mg die x 2w + Omeprazole 20 mg die x 2 w) + narcotic for emergency pain relief only **I2:** Multimodal analgesic regiment + narcotic as needed	No standing dose regimen (acetaminophen 500 mg QID PRN x 4w + Oxycodone q. 4h PRN + tramadol 50 mg q 6 hours PRN)	Cumulative MED	*1 month:* I1: mean difference: **-0.77**, *p* < 0.001); I2: **-0.30**, *p* = 0.04 compared to C I1: mean difference: **-0.46**, *p* = 0.002 compared to I2.
								Proportion of patients who received opioid refill (%)	*3 months*: I1: 10.50%; I2: 6.50%; C: 15.60%, No significance test
								Proportion of patients using opioids	I1: 0.00%; I2: 1.30%; C: 2.60%, No significance test
Hannon 2019 USA [68]	Randomized controlled trial	304	65	54%	THA or TKA	Prescriptions of acetaminophen, meloxicam, gabapentin, tramadol, and **30** pills of 5 mg OxyIR (oxycodone) as a second breakthrough pain medication	Idem as experimental group and **90** pills of 5 mg OxyIR (oxycodone)	Cumulative MED	*1 month :* I: 456.70; C: 455.60, *p* = 0.980 *3 months*: I: **777.10**; C: 1089.70, *p* < 0.001
								Proportion of patients who received opioid OxyIR refill(s) (%)	*3 months:* I: 26.70%; C: **10.50%**, *p* < 0.001
Padilla 2019 USA [84]	Retrospective cohort	669	65	58%	THA	Opioid sparing pain management protocol (intravenous acetaminophen, periarticular injection of liposomal bupivacaine, pre-emptive analgesia in postoperative period)	Before implementation of the opioid sparing protocol	Cumulative MED	*3 months:* I: **13.90**; C: 80.10, *p* < 0.001
Tan 2018 Australia [58]	Prospective cohort	230	64	66%	THA	ERAS program (multimodal analgesia, early mobilization with physiotherapy)	Before ERAS implementation	MED/day	*6 weeks*: I: 0.00; C: 0.00, *p* > 0.99
								Proportion of patients using opioids (%)	The proportion of patients with zero MED consumption at week 6 increased from **56.60% to 80.00%** (RR 1.34, 95% CI 1.13-1.58).
Dasa 2016 USA [56]	Retrospective cohort	100	38	70%	TKA	Administering perioperative cryoneurolysis and multimodal analgesics regimen	Multimodal analgesics regimen alone.	Cumulative MED	*3 months*: I: **2,069.12**; C: 3,764.42, *p* < 0.0001
**Surgical**									
Bovonratwet 2021 USA [111]	Retrospective cohort	611	63	81%	THA	Direct anterior approach	Posterior approach	MED	*No data available on the amount of prescribed or consumed opioids*
								Proportion of patients who required a refill	*3 months:* I: 14.77%; C: 20.73: *p* = 0.077 I relative to C: relative risk = 0.95, 95% CI 0.55-1.64, *p* = 0.864
Varady 2021 USA [112]	Retrospective cohort	92, 506	57	52%	TJA	Outpatient (no overnight stay)	Inpatient	Proportion of new opioid persistent use (patient still filling opioid prescriptions >90 days postop)	*3 months:* I: **8.20**; C: 10.60 *p* < 0.001 OR, **1.21**; 95% CI 1.11-1.32; *p* < 0.001
Walega 2019 USA [61]	Randomized controlled trial	68	66	60%	TKA	Genicular nerve radiofrequency aoublation	Sham procedure: simulated GN-RFA using identical supplies and devices	MED/day	*6 months:* I: 0.00; C: 0.00, *p* = 0.58
Verla 2018 USA [60]	Retrospective cohort	46	58	54%	Spine surgery	Transforaminal lumbar interbody fusions	Direct lateral lumbar interbody fusions	Postoperative opioids duration in months	*All level*: I: 5.20; C: 4.80, *p* = 0.82 *L4-L5 only*: I: 4.30; C: 3.14, *p* = 0.5
Della Valle 2010 USA [59]	Randomized controlled trial	72	63	68%	THA	Mini-incision approach	2 incisions approach	MED/day	*6 weeks:* I: 1.30; C: 1.40, *p* = 0.79
**Alternative**									
Collinsworth 2019 USA [54]	Randomized controlled trial	40	20	22%	Shoulder surgery (military population)	Usual care and BFA (semipermanent acupuncture needles emplaced on the subjects’ ears for 3–5 days within 24 hours after shoulder surgery. BFA was reapplied, as needed, up to 6 weeks post-surgically)	Usual postsurgical care (include surgery specific protocols, therapeutic modalities and prescribed/ over-the-counter pain medications	Daily opioid use	*6 weeks:* *m*ean difference: 3.75, 95% CI -3.335-10.825, *p* = 0.29
**Psychological**									
Hanley 2021 USA [113]	Randomized controlled trial	118	65	62%	THA, TKA	One 20 minutes session of mindfulness of breath (I1) or mindfulness of pain (I2) 3 weeks preop	One 20 minutes session of cognitive-behavioral pain psychoeducation (C)	Opioid use	*Until 28 days postoperatively* Both MoB and MoP decreased postoperative opioid use relative to C, F(8, 83) = **16.66**, *p* < 0.001
Hah 2020 USA [114]	Randomized controlled trial	104	66	52%	THA, TKA	Motivational interviewing and guided opioid tapering support added to usual care (phone call weekly for postoperative weeks 2-7 and monthly up to 1 year or to opioid cessation)	Usual care + standardized verbal and written instructions on the proper analgesic use of opioids before surgery	Time to base line opioid use return (days)	I: **34.60**; C: 67.80, HR **1.62**; 95% CI 1.06- 2.46; *p* = 0.03
								Proportion of patients using opioids at 3 months	Overall: I: 2.70%; C:2.00, *p* > 0.05 Opioid naïve: I: 2.70%; C: 9.50%, *p* > 0.05 Preoperative user: I: 8.30%; C: 23.10%, *p* > 0.05
								Proportion of patients using opioids at 6 months	Overall: I:0.00%; C:5.50%, *p* > 0.05 Opioid naïve: I: 0,00%; C: 2,40%, *p* > 0.05 Preoperative user: I: 0.00%; C: 15.40%, *p* > 0.05
								Time to postoperative opioid cessation (days)	I: 41.1; C: 76.4 HR 1.57; 95% CI 1.01- 2.44; *p* = 0.05
								Proportion of opioid cessation	I: 91.80%; C: 87.3%, *p* = 0.5
Dindo 2018 USA [95]	Randomized controlled trial	75	63	6%	Orthopedic surgeries (no trauma)	Acceptance and Commitment Therapy (ACT) and treatment as usual	Treatment as usual (a nurse-led patient education class + analgesia with opioids +/- nonopioids, anticonvulsants or anxiolytics regular or as need. Discharge combination of an opioid and acetaminophen	Time to opioid cessation (days)	I: 42.50; C: 51.00; HR 1.44, 95% CI 0.74-2.78
								Proportion of patients using opioids	*At 7 weeks*: I: 29.00%; C: 52.00%, OR= 0.39; 95% CI 0.14-1.08

*Abbreviations*: *ACDF* anterior cervical discectomy and fusion, *ACLR* Anterior cruciate ligament reconstruction, *ARCR* arthroscopic rotator cuff repair, *BFA* Battlefield Acupuncture, *C* Control group, *COU* Chronic opioid use, *CTR* carpal tunnel release, *ERAS* Enhanced recovery after surgery, *FAI* femoroacetabular impingement, *FNB* Femoral Nerve Block, *HR* Hazard ratio, *I* Intervention group, *LIA* Local Anaesthetic Infiltration, *MED* Morphine equivalent dose, *OR* Odds ratio, *ORIF* open reduction and internal fixation, *RANDOMIZED CONTROLLED TRIAL* Randomized control trial, *RCR* rotator cuff repair, *TAA* total ankle arthroplasty, *THA* total hip arthroplasty, *TKA* Total knee arthroplasty, *TSA* Total Shoulder Arthroplasty

^a^Confidence intervals were described when available in the original studies

^b^Tier = Number of pills prescribed according to the type of surgery

The sample sizes ranged from 40 [54] to 120,080 [73] participants (mean = 5, 094, median = 230) with an average age between 20 [54] and 75 [93] years, but greater than 55 years in the majority of studies (*n* = 49). More than 60% of the studies (*n* = 41) had more than 50% of females (range from 1.0 to 81.0%). Most of the selected studies (62.1%) focused on the elective orthopaedic surgery population who underwent procedures to the limbs [50, 51, 54, 56–59, 61, 62, 64, 65, 67, 68, 70–74, 76, 78, 82, 84, 88–92, 95, 102–114]. The remaining studies targeted trauma populations (18.2%) [5, 63, 66, 75, 87, 93, 94, 96, 98–101] or a mix of trauma and elective orthopaedic surgical patients (12.1%) [52, 53, 55, 77, 79, 80, 86, 97], and patients who underwent spine surgery performed by orthopaedic or neurosurgeons (7.6%) [60, 69, 81, 83, 85].

Risk factors for chronic opioid use (e.g., previous opioid use, benzodiazepine use, substance abuse, mental health disorder, chronic pain) were measured in close to 70% of studies [5, 50, 51, 56–58, 61, 63, 64, 67, 68, 72–74, 78, 79, 82–89, 92–95, 97–100, 102–114] (Supplemental Digital File 4: Risk factors for chronic opioid use in included studies by types of strategies). The main risk factors involved were depression/anxiety or associated medication use [51, 53, 56, 57, 63, 64, 72–74, 78, 79, 82, 83, 85–89, 92, 97, 100, 102, 104, 107, 109, 112, 114], and prior opioid use [5, 53, 57, 58, 61, 63, 64, 67, 68, 73, 74, 78, 82, 83, 85–89, 95, 97–100, 102–104, 107–109, 111, 113, 114]. Amongst studies that included patients at risk for chronic opioid use, nearly 60% [51, 53, 63, 64, 72, 74, 78, 82, 83, 85, 89, 94, 95, 97–100, 102–104, 106, 107, 109, 110, 112–114] included a sample with a risk ≥25%, but only 20% [74, 78, 83, 95, 100, 104, 107, 110, 113] included a sample with a risk of ≥50% or more.

As described in Table 1: Study Characteristics, Description of Strategies and Outcomes, selected studies were divided in seven categories, according to the type of strategy assessed. Among *system-based* strategies, we identified 22 studies on hospital-based protocols to limit or improve opioid prescriptions (*n* = 13) [77–83, 89, 90, 92, 96, 97, 102] or formal government regulation in some U.S. states to limit opioid prescriptions (*n* = 9) [85–88, 91, 93, 103–105]. Among *pharmacological* strategies, we identified 18 studies [62–76, 94, 98, 99, 106], which mainly focused on the effect of anesthetic agents administered through regional anesthesia (*n* = 8) [62, 65, 70, 73, 74, 94, 98, 99] and the impact of medication on the central nervous system [63, 66, 67, 69] (*n* = 4) and opioid use. The *educational* strategies comprised seven studies on strategies aimed at providing patient information on the adequate use of opioids (*n* = 6) [5, 50, 51, 53, 100, 107] or aimed as a reminder to professionals on opioid prescribing guidelines (*n* = 1) [52]. The *multimodal* strategies included eight studies testing strategies on multiple analgesic regimens or a combination of pharmacological and non-pharmacological strategies [56–58, 68, 84, 101, 108, 110] and a transitional pain service [133]. Finally, five studies focused on the effect of *surgical techniques* (e.g., different surgical approaches [59–61, 111]) or inpatient vs. outpatient surgery [112]; two on an *alternative* pain management strategy (i.e., acupuncture) [54, 55], and three on a *psychological* strategy (i.e., Acceptance and Commitment Therapy [95], motivational interviewing [114] or mindfulness [113].

We identified three outcomes related to opioid use. The most commonly measured outcome was the quantity of opioids in morphine equivalent doses (MEDs) (*n* = 44) [53, 55–59, 61, 63, 68, 70, 72, 74–77, 79–87, 89–93, 96–108, 110, 111], generally measured at 6 weeks or 1, 3 and 6 months. The proportion of patients using opioids was the second most frequently used outcome measure in the selected studies (*n* = 25) [5, 53, 57, 58, 62–65, 67, 69, 72, 73, 78, 81, 86, 94, 96, 100, 101, 105, 107, 109, 112, 114, 134] and this evaluation was often conducted at 3 (*n* = 13) [5, 53, 57, 62, 63, 65, 72, 81, 94, 100, 105, 112, 114] and 6 months (*n* = 6) [64, 67, 78, 101, 114]. The least frequently measured outcome was the proportion of patients who received an opioid prescription refill (*n* = 18) [57, 68, 79–81, 83, 89–92, 96, 97, 102, 103, 105, 108, 110, 111] at 1 and 3 months. None of the studies measured illicit opioid use or opioid diversion (i.e., opioid diverted from siblings who have legitimate prescriptions). Data came from patient records or clinical-administrative databases (*n* = 27) [5, 60, 73, 74, 78–81, 83–89, 91, 94, 98, 101–105, 108, 110, 112, 114], or a combination of these methods, and from patient self-report (*n* = 17) [53, 55, 58, 62, 63, 65, 68, 71, 72, 77, 82, 90, 96, 100, 107, 109, 113] in many studies. The remaining studies measured outcomes from self-reported data only (*n* = 5) [50, 54, 67, 69, 95] or did not clearly describe the data source (*n* = 17) [51, 52, 56, 57, 59, 61, 64, 66, 70, 75, 76, 92, 93, 97, 99, 106, 111]. Regarding the proportion of trauma and orthopaedic surgical patients still using opioids at follow-up, the proportion of patients using opioids at 3 months as reported in some studies varied from 12 to 30% in trauma patients [63, 86, 100] and from 10 to 50% in elective orthopaedic surgical patients [67, 78, 95, 107, 112] presenting risk factors for chronic opioid use. One study also reported proportions of almost 70% for patients with a history of chronic opioid use at 12 months in the context of elective orthopaedic surgery [73]. Two studies concerning trauma populations documented proportions of 20 to 40% for patients with no documented risk factors at 3 months [5, 94] and the proportion decreased to 15% at 6 months [94]. For other studies conducted mostly in non-trauma patients without or with minimal risk factors (≤ 25% of the sample), the proportion varied from 0% (intervention group only) to 25% at 3 months [57, 65, 105, 107, 109, 114].

As shown in Table 2: Study Characteristics, Description of Strategies and Outcomes, most guidelines came from the U.S. (*n* = 13) [47, 48, 115, 118–123, 126, 128, 130, 131]. They provided recommendations to prevent chronic opioid use in the following populations: surgery in general (20%) [48, 116, 117, 123], orthopaedic surgery (35%) [118, 124, 125, 127, 128, 130, 132], trauma (25%) [47, 122, 126, 129, 131], a combination of trauma and orthopaedic surgery (10%) [119, 120] as well as general and orthopaedic surgery (5%) [115], or patients with acute pain (5%) [121]. Guideline recommendations were classified according to the following categories: system-based, pharmacological, educational and multimodal.

**Table 2:** Recommendations from guidelines, their level of evidence and their strength

Author (Sponsor), year, country	Population	Recommendations	Level of evidence
**System-Based**			
Edwards (ASER, POQI), 2019, USA [117]	Patients on preoperative opioids	Patients should be assessed for risk factors for persistent opioid use prior to the initiation of opioid therapy and during therapy to develop and coordinate the pain treatment plan with the health care team.	Recommended (GRADE)
Kent (ASER, POQI, 2021, USA [115]	Surgery		Suggested
Clarke, 2020, Canada [116]	Surgery		Expert consensus
Trexler, 2020, USA [131]	TBI		
Soffin, 2017, USA [118]	Orthopedic surgery		
Washington State AMDG, 2015, USA [120]	All patients		
The committeee on trauma of the ACS, 2020, USA [126] ^a^	Trauma		No level of evidence
Chou (APS, ASRA, ASA), 2016, USA [48]	Surgery	Clinicians should conduct a preoperative evaluation to guide the intraoperative pain management plan. It should include: assessment of medical and psychiatric comorbidities, concomitant medications, history of chronic pain, substance abuse, and previous postoperative treatment regimens and responses.	Strong recommendation, low-quality evidence
Soffin, 2017, USA [118]	Orthopedic surgery	Opioid tolerance should be diagnosed preoperatively. Referral to an addiction specialist should be made in the presence of opioid-tolerance.	Expert consensus
Mai, 2015, USA [119]	Musculoskeletal injuries		
Hsu, 2019, USA [46]	Trauma	Doses of prescribed controlled substances should be verified via the relevant state Prescription Drug Monitoring Program (PDMP), or by contacting the original prescriber or dispensing pharmacist.	Strong recommendation, low-level of evidence
Soffin, 2017, USA [118]	Orthopedic surgery		Expert consensus
Mai, 2015, USA [119]	Musculoskeletal injuries		
Washington State AMDG, 2015, USA [120]	All patients		
The committee on trauma of the ACS, 2020, USA [126]	Trauma		No level of evidence
Chou (APS, ASRA, ASA), 2016, USA [48]	Surgery	Facilities in which surgery is performed should provide clinicians with referral options to a pain specialist for patients with inadequately controlled postoperative pain or at high risk of inadequately controlled postoperative pain (e.g. opioid-tolerant, history of substance abuse)	Strong recommendation, low-quality evidence
Clarke, 2020, Canada [116]	Surgery		Expert consensus
Sodhi, 2020, USA [130]	TJA		
Soffin, 2017, USA [118]	Orthopedic surgery		
Mai, 2015, USA [119]	Musculoskeletal injuries		
The committee on trauma of the ACS, 2020, USA [126]	Trauma	If pain persists beyond 3 months, or if opioid misuse by patient is suspected, the patient should be referred to a transitional/chronic pain clinic or pain management specialist.	No level of evidence
		The trauma center should provide a pain management service or resources to act as an expert consultant within the trauma service.	
Edwards (ASER, POQI), 2019, USA [117]	Patients on preoperative opioids	The patient’s outpatient opioid prescriber should be identified and be contacted to anticipate discharge needs and to coordinate postoperative opioid tapering.	Recommended (GRADE)
The committee on trauma of the ACS, 2020, USA [126]	Trauma		No level of evidence
Hsu, 2019, USA [46]	Trauma	For patients using illicit opioids, or patients misusing prescription opioids, follow-up should be coordinated with acute pain services (or addiction medicine or psychiatry depending on resources) for inpatients, and with the patient’s prescriber for outpatients, to ensure that there is only 1 prescriber for patients on medication-assisted therapy.	Strong recommendation, moderate-quality evidence
The committee on trauma of the ACS, 2020, USA [126]	Trauma		No level of evidence
Hsu, 2019, USA [46]	Trauma	Prescribers, to the extent possible, should develop and/or support the implementation of a support system to inform clinical decisions regarding opioid prescription in the electronic medical record.	Strong recommendation, low-level of evidence
The committee on trauma of the ACS, 2020, USA [126]	Trauma		No level of evidence
Kent (ASER, POQI), 2021, USA [115]	Surgery	Persistent postoperative opioid use occurs when a patient interacts with numerous health care providers and institutions. Addressing system-based characteristics may be more instrumental in tapering persistent opioid use than clinical decision making. Public health initiatives, policies, and legislation at the local, state, and federal levels aimed at safe opioid prescribing should be evaluated with subsequent recommendations for further improvements that target all health care system components.	Strongly recommended
Trexler, 2020, USA [131]	TBI		Expert consensus
U.S. Department of Health ad Human Services (Task Force), 2019, USA [121]	All patients	Complex opioid and non-opioid management should be reimbursed with the time and resources required for patient education; safe evaluation; risk assessment; re-evaluation; and integration of alternative and non-opioid modalities.	Expert consensus
**Pharmacological - Opioid Prescription Practices**			
Wainwright (ERAS Society), 2020, UK [132]	TJA	Add opioids only in the setting of suboptimal analgesia after first-line administration of nonopioid options or when the benefits outweigh the risks	Strongly recommend, High level of evidence
Edwards (ASER, POQI), 2019, USA [117]	Patients on preoperative opioids		Recommended (GRADE)
Anger (PROSPECT), 2021, USA [127]	TJA		Expert consensus
Trexler, 2020, USA [131]	TBI,		
Franz (DMGP), 2019 Germany [129]	SCI		
U.S. Department of Health ad Human Services (Task Force), 2019, USA [121]	All patients		
			No level of evidence
The committee on trauma of the ACS, 2020, USA [126]	Trauma		
Hsu, 2019, USA [46]	Trauma	The prescriber should use the lowest opioid effective dose for the shortest time period possible.	Strongly recommended, high-quality evidence
Edwards (ASER, POQI), 2019, USA [117]	Patients on preoperative opioids		Recommended (GRADE)
Trexler, 2020, USA [131]	TBI		Expert consensus
U.S. Department of Health ad Human Services (Task Force), 2019, USA [121]	All patients		
Soffin, 2017, USA [118]	Orthopedic surgery		
Washington State AMDG, 2015, USA [120]	All patients		
Edwards (ASER, POQI), 2019, USA [117]	Patients on preoperative opioids	The prescriber should avoid opioid dose escalation.	Recommended (GRADE)
Washington State AMDG, 2015, USA [120]	All patients		Expert consensus
The committee on trauma of the ACS, 2020, USA [126]	Trauma	Have a protocol for safe de-escalation of analgesics as quickly as possible.	No level of evidence
The committee on trauma of the ACS, 2020, USA [126]	Trauma	Promptly investigate the cause of increasing pain rather than responding by increasing the analgesic dose or adding new medications	No level of evidence
Hsu, 2019, USA [46]	Trauma	Prescribe precisely - Commonly written prescriptions with ranges of dose and duration can allow tripling of daily dose to levels consistent with adverse events.	Strongly recommended, low-level evidence
Hsu, 2019, USA [46]	Trauma	Avoid long-acting opioids in the acute phase.	Strongly recommended, moderate-quality evidence
Trexler, 2020, USA [131]	TBI		Expert consensus
Hsu, 2019, USA [46]	Trauma	Benzodiazepines should not be prescribed in conjunction with opioids because of the significant risks posed by inconsistent sedation and the potential for misuse.	Strongly recommended, high-quality evidence
Trexler, 2020, USA [131]	TBI		Expert consensus
Clarke, 2020, Canada [116]	Surgery	Patients should receive a prescription based on their opioid consumption in the hospital during the previous 24 hrs that should be written during the discharge process.	Expert consensus
The committee on trauma of the ACS, 2020, USA [126]	Trauma		No level of evidence
The committee on trauma of the ACS, 2020, USA [126]	Trauma	Discharge prescriptions should separate opioids and nonopioid analgesics to make opioid tapering easier.	No level of evidence
Washington State AMDG, 2015, USA [120]	All patients	Strongly consider tapering the patient off opioids as the acute pain episode resolves.	Expert consensus
Clarke, 2020, Canada [116]	Surgery	The prescription for opioid-containing tablets should have an expiry date of 30 days from the date of discharge	
Washington State AMDG, 2015, USA [120]	All patients	A part-fill or prescription refill should be given to patients with an expected moderate or long-term recovery to reduce the number of opioid tablets distributed at one time.	Expert consensus
Hsu, 2019, USA [46]	Trauma	The prescription and continued use of opioids should be based on expected functional recovery, pain, opioid use and adverse events. Complete and regular evaluations are therefore necessary.	Strong recommendation, low-quality evidence
Chou (APS, ASRA, ASA), 2016, USA [48]	Surgery		
Clarke, 2020, Canada [116]	Surgery		Expert consensus
Trexler, 2020, USA [131]	TBI		
Mai, 2015, USA [119]	Musculoskeletal injuries		
U.S. Department of Health ad Human Services (Task Force), 2019, USA [121]	All patients		
Washington State AMDG, 2015, USA [120]			
The committee on trauma of the ACS, 2020, USA [126]	Trauma		No level of evidence
Soffin, 2017, USA [118]	Orthopedic surgery	The patient has to be physically present when the initial prescription for a controlled substance is made. No new prescriptions are made or refilled if the patient has not been seen and examined within the prior 30 days.	Expert consensus
Clarke, 2020, Canada [116]	Surgery	Patients should be discharged with a prescription for the following adjunct pain medications, unless contraindicated: Acetaminophen, NSAIDS	Expert consensus
Fillingham (AAHKS, ASRA, AAOS, Hip society, Knee society), 2020, USA [127]	TJA		
Sodhi, 2020, USA [130]			
Trexler, 2020, USA [131]	TBI		
U.S. Department of Health ad Human Services (Task Force), 2019, USA [121]	All patients		
Washington State AMDG, 2015, USA [120]			
The committee on trauma of the ACS, 2020, USA [126]	Trauma		No level of evidence
Anger (PROSPECT), 2021, USA [127]	TJA	Postoperative NSAID are recommended for their analgesic and opioid-sparing effect.	High-quality evidence
Wainwright (ERAS Society), 2020, UK [132]	TJA		Strong recommadation, moderate – high level of evidence
Fischer (PROSPECT), 2008, UK [124]	TKA		Low level of evidence
Ftouh (NICE), 2011, UK [123]	Hip fracture	NSAID should not be used for pain management after a hip fracture because of their poor risk to benefit ratio	Expert consensus
**Educational**			
Hsu, 2019, USA [47]	Trauma	Health service departments should support opioid education efforts for prescribers and patients.	Strongly recommended, moderate-quality evidence
Anger (PROSPECT), 2021, USA [127]	TJA	Patients should be provided education in the pre-operative period.	High-quality evidence
Wainwright (ERAS Society), 2020, UK [132]	TJA		Strong recommendation, low level of evidence (GRADE)
Clarke, 2020, Canada [116]	Surgery	Patients should receive written and verbal information prior to discharge on the safe storage and disposal of unused opioids.	Expert consensus
Trexler, 2020, USA [131]	TBI		
Hsu, 2019, USA [47]	Surgery	Clinicians should provide education to all patients and / or family and/or primary caregivers: • On treatment options for pain management, the plan and goals for pain management and the pain treatment plan, including analgesic tapering after hospital discharge. • To fill the prescription only if their pain is not adequately managed with other therapies or if they are having difficulty completing activities of daily living secondary to pain. • On the risks and benefits of alternatives to chronic opioid therapy.	Strong recommendation, low-quality evidence
Clarke, 2020, Canada [116]	Surgery		Expert consensus
Trexler, 2020, USA [131]	TBI		
U.S. Department of Health ad Human Services (Task Force), 2019, USA [121]	All patients		
Washington State AMDG, 2015, USA [120]			
The committee on trauma of the ACS, 2020, USA [126]	Trauma		No level of evidence
Chou (APS, ASRA, ASA), 2016, USA [48]	Surgery	Patients chronically prescribed opioids before surgery should be instructed: • On how to taper opioids to their target maintenance dose • On who will prescribe controlled substances after surgery and discharge from hospital.	Strong recommendation, low-quality evidence
Soffin, 2017, USA [118]	Orthopedic surgery		Expert consensus
U.S. Department of Health ad Human Services (Task Force), 2019, USA [121]	All patients	Use apps for biopsychosocial treatments to inform physicians, providers, and patients on evidence-based and effective pain management treatments for various chronic pain syndromes more effectively.	Expert consensus
**Multimodal**			
Chou (APS3, ASRA4, ASA5), 2016, USA [48]	Surgery	Nonopioid therapy should be the first-line of treatment and multimodal analgesia should be used as opposed to opioid monotherapy for pain control. Therapies can be pharmacological or nonpharmacological.	Strong recommendation, high-quality evidence
Wainwright (ERAS Society), 2020, UK [132]	TJA		
Hsu, 2019, USA [47]	Trauma		Strong recommendation, moderate-quality evidence
Edwards (ASER, POQI), 2019, USA [117]	Patients on preoperative opioids		Strongly recommended(GRADE)
Galvagno (EAST, TAS), 2016, USA [122]	Blunt thoracic trauma		Conditionally recommended, very-low quality evidence
Wu (ASER), 2019, USA [123]	Surgery		Expert consensus
Wu (ASER), 2019, USA [123]	Surgery	Patients should be discharged home with a comprehensive multimodal analgesia care plan aiming to minimize or avoid post-discharge opioid use.	Expert consensus
Chou et al. (APS, ASRA, ASA), 2016, USA [48]	Surgery	Health professionals should consider gabapentin or pregabalin as components of multimodal analgesia.	Strong recommendation, moderate-quality evidence
U.S. Department of Health ad Human Services (Task Force), 2019, USA [121]	All patients	For neuropathic pain, as first-line therapy, consider anticonvulsants (gabapentin, pregabalin, carbamazepine, oxcarbazepine), SNRIs (duloxetine, venlafaxine), TCAs (nortriptyline, amitriptyline), and topical analgesics (lidocaine, capsaicin).	Expert consensus
Washington State AMDG, 2015, USA [120]			
Chou (APS, ASRA, ASA), 2016, USA [48]	Surgery	Health professionals should consider ketamine as a component of multimodal analgesia in adults.	Weak recommendation, moderate-quality evidence
Fischer (PROSPECT), 2008, UK [124]	TKA	Cooling and compression techniques should be used for postoperative analgesia, based on limited procedure-specific evidence, for a reduction in pain scores and analgesic use.	Low level of evidence
Chou et al. (APS, ASRA, ASA), 2016, USA [48]	Surgery	Health professionals should consider transcutaneous electrical nerve stimulation (TENS) as an adjunct to other pain management strategies.	Weak recommendation, moderate-quality evidence
Washington State AMDG, 2015, USA [120]	All patients	In addition to medication, therapies should include physical activation and behavioral health interventions (such as cognitive behavioral therapy, mindfulness, coaching, patient education, and self-management).	Expert consensus
Hsu et al., 2019, USA [47]	Trauma		Strong recommendation, moderate-quality evidence
U.S. Department of Health ad Human Services (Task Force), 2019, USA [121]	All patients	Consider complementary and integrative health approaches, including acupuncture, mindfulness meditation, movement therapy, art therapy, massage therapy, manipulative therapy, spirituality, yoga, and tai chi, in the treatment of acute and chronic pain, when indicated.	Expert consensus
The committee on trauma of the ACS, 2020, USA [126]	Trauma	Nonpharmacologic pain management strategies are recommended as adjuncts for pain and anxiety management in trauma to minimize opioid use and chronic pain development	No level of evidence

*Abbreviations*: *ACS* American College of Surgeons, *AMDG* Agency Medical Directors’ Group, *APS* American Pain Society, *ASA* American Society of Anesthesiologists, *ASER* American Society for Enhanced Recovery, *ASRA* American Society of Regional Anesthesia and Pain Medicine, *EAST* Eastern Association for the Surgery of Trauma, *GRADE* Grading of Recommendations Assessment, Development, and Evaluation, *NICE* National Institute for Health and Clinical Excellence, *POQI* Perioperative Quality Initiative, *TAS* Trauma anesthesiology society, *Task Force* Pain Management Best Practices Inter-agency Task Force, *TJA* Total joint. Arthroplasty, *TKA* Total knee arthroplasty

^a^This source does not describe any method for classifying the level of evidence of recommendations

### Evidence on preventive strategies

#### System-based

As described in Table 1: Study Characteristics, Description of Strategies and Outcomes, this category contains 19 retrospective [77–83, 85–89, 91–93, 97, 102–105] and three prospective cohort studies [77, 90, 96], whose comparators were all pre-intervention data.

Most studies on hospital-based and government regulation initiatives limiting the prescription of opioids showed a significant decrease in MED taken by opioid-naïve and non-opioid naïve patients at 1 month after trauma [86, 87, 93] and elective orthopeadic surgery [85, 88, 90, 91, 104], and mainly in opioid-naïve patients up to 3 months in these two populations [82, 86, 92, 102–105]. Also, regulation on prescription limits led to mixed results on opioid refills, with a significant decrease [80, 82, 103, 105] or increase [88, 90, 91, 102] after trauma and elective orthopaedic surgery. Strategies related to the implementation of prescription guidelines did not lead to a significant decrease in MED, opioid refills or the proportion of patients using opioids after trauma at 1 month [77, 96, 97]. However, strategies based on individualized opioid tapering protocols, which were not evaluated in the context of trauma, led to a significant reduction in opioid use in MED 6 weeks [89] and 6 months after spine surgery [83]. About a third of patients were using opioids preoperatively in these studies [83, 89]. It is interesting to note that most studies performed in elective orthopaedic surgical context had more than 25% of patients at high risk of chronic opioid use (i.e., prior opioid use, alcohol abuse or psychological comorbidities) [78, 82, 83, 85, 89], all of which demonstrated at least one statistically significant result favouring the group that received a preventive strategy.

System-based strategies were also frequently addressed in practice guidelines (Table 2: Study Characteristics, Description of Strategies and Outcomes). However, recommendations were mostly based on expert consensus or low-quality evidence. Guidelines strongly emphasized the importance of an early assessment of patients’ risk factors in order to plan for the required follow-up [48, 115–117, 121, 131]. Similarly, communication between professionals was recommended in order to establish the required follow-up, to avoid multiple prescribers, and to refer patients to specialized resources in a timely manner after trauma and orthopaedic surgery, particularly those misusing opioids or with a history of substance abuse [47, 115–119, 130].

#### Pharmacological

This category includes ten RCTs [62, 65–67, 69–71, 75, 76, 106], six retrospective [72–74, 94, 98, 99] and two prospective cohort studies [63, 64] (Table 1: Study Characteristics, Description of Strategies and Outcomes). Most studies used a placebo (*n* = 9) [62, 66, 67, 69, 71, 74–76, 106] or no intervention (*n* = 7) [63, 64, 72, 73, 94, 98, 99] as comparators. Studies comparing the use of regional anesthesia to general anesthesia in trauma patients, showed an increase in MED at 3 months post-injury [98, 99]. One study evaluating the impact of recreational cannabis use during the recovery of trauma patients showed a significant increase in MED at 6 months and in the duration of opioid use, compared to patients who never used this drug [63]. Among the studies that analyzed the effect of nerve blocks as a preventive strategy [62, 65, 70, 73, 74, 94], compared to usual care or placebo, none showed a significant decrease in MED [70, 74] or in the proportion of opioid-naïve and non-opioid naïve patients using opioids [62, 65, 73, 94] in the trauma or orthopaedic surgical populations at 3 months and beyond. Likewise, drugs with an impact on the central nervous system (e.g., gabapentinoids, antidepressants) [66, 67, 69] were not significantly associated with a reduction in opioid use in patients with burn injury [66] or who underwent spine [69] or elective orthopaedic surgery [67]. Three RCTs [71, 76, 106] on the regular use of postoperative NSAIDs compared to placebo showed a significant decrease in opioid use up to 12 months after an elective orthopaedic surgical procedure (i.e., total knee arthroplasty or shoulder surgery) regardless of whether or not opioids were taken prior to surgery. Beta blockers have also been associated with a reduction in MED and in the proportion of patients using opioids at 1 month after an elective orthopaedic surgery in a retrospective study conducted in a population with a large proportion of patients with a history of depression [72].

Many guidelines (Table 2: Study Characteristics, Description of Strategies and Outcomes) emphasized the safety of opioid prescriptions [47, 116–118, 120, 121, 125, 127, 129, 131, 132] in trauma and surgical populations. Those guidelines recommended using opioids only when necessary [117, 118, 127, 129, 131, 132], at the lowest effective dose [47, 117, 118, 131], avoiding dose escalation [117, 120], and using opioids for the shortest period of time possible [47, 118, 131]. These recommendations were associated with moderate to high levels of evidence in trauma [47] and in patients on preoperative opioid use [117]. The use of NSAIDs as a strategy to limit long-term opioid use was rated as high-quality evidence in one guideline on elective orthopaedic procedures [125]. Guidelines also specified that opioid prescriptions must be tailored to the patient’s condition [47, 48, 116, 119, 121, 126, 131]. However, these recommendations were mainly based on expert consensus.

#### Educational

This category comprises three RCTs [50, 51, 53, 100, 107] and two retrospective cohort studies [5, 52], the majority of which used standard educational programs [50–53, 100, 107] or no educational intervention [5] as comparators (Table 1: Study Characteristics, Description of Strategies and Outcomes). Many studies evaluating educational strategies reported positive outcomes after trauma and orthopaedic surgery. MED was measured in two studies [53, 100] and favoured (one study with significant result [53] and one small pilot study without significant results [100]) the group of patients who received a formal education program compared to usual care, at 6 weeks and 3 months after an injury. These outcomes were achieved despite the presence of more than 25% of patients at high risk (i.e., history of substance abuse, psychological comorbidities, opioid use before the injury) for long-term opioid use in the study population. However, another study conducted in elective orthopaedic surgery showed a significant decrease in the proportion of opioid dependence (i.e., 6 opioid prescriptions from the date of surgery) only in opioid-naïve patients [107]. Time to opioid cessation was measured in two studies [50, 53] and was significantly lower in patients who received educational strategies after traumatic injuries [53] and elective orthopaedic surgery [50]. Finally, an educational program for hand surgeons led to a 20% significant decrease in prescribed opioids at 3 months [52].

Guidelines also provided recommendations on educational strategies for patients and health care professionals [47, 48, 116, 118, 121, 127, 131, 132] after trauma and surgery, but the majority were based on expert consensus or lower quality evidence (Table 2: Study Characteristics, Description of Strategies and Outcomes). These recommendations focused on educating patients and/or families about the risks and benefits of opioids [121, 131], different pain management methods to limit opioid use [116, 118, 121], and information on storing and returning medications after surgery [116, 131]. They also provided advice on using a monitoring and tapering opioids plan [48, 118].

#### Multimodal

This category includes two RCTs [57, 68], two prospective [58, 110] and five retrospective cohort study [56, 84, 101, 108, 109] comparing different types of multimodal regimens to usual treatment (Table 1: Study Characteristics, Description of Strategies and Outcomes). Although less numerous, multimodal strategies were also associated with several favourable outcomes after trauma and elective orthopaedic surgery. For example, studies on multimodal analgesic regimens with minimal opioid use showed significant reductions in MED but not in the proportion of opioid users at 6 months after trauma [101] compared to patients who received a regimen mainly based on opioids. It should be noted that risk factors for opioid use were not specified in this study and that only 3% of patients were still taking opioids in both groups at 6 months. Still, for the elective orthopaedic population, a significant decrease in MED was reported up to 3 months in opioid-naïve and non-opioid naïve patients [57, 68, 84, 110]. Moreover, the proportion of opioid-free patients at 6 weeks increased significantly for those who received an enhanced recovery after surgery (ERAS) program, consisting of pharmacological and non-pharmacological strategies for pain management, compared to those who did not after an elective orthopaedic surgery [58]. Such results were also observed after the implementation of a transitional multidisciplinary service in opioid-naïve and non-opioid naïve veterans [109]. A large proportion of this population had a history of mental health disorders. Finally, studies demonstrated a significant reduction in MED at 6 weeks to 3 months by adding cryoneurolysis to multimodal analgesic regimen in an elective orthopaedic surgery population without risk factors for chronic opioid use [56, 108].

Most guidelines [47, 48, 116–118, 120, 122, 123, 125, 128, 130–132] recommended a multimodal analgesia plan (i.e., acetaminophen, NSAIDs) as a first line of treatment to limit opioid use in trauma and surgical patients, based on moderate to high quality evidence (Table 2: Study Characteristics, Description of Strategies and Outcomes). A few guidelines [48, 120, 121] also recommend the addition of anticonvulsants (e.g., pregabalin) under specific conditions, such as neuropathic pain. Several guidelines also propose non-pharmacological strategies [116, 117], including complementary and integrative health approaches (e.g., acupuncture, mindfulness meditation) [121], transcutaneous electrical nerve stimulation (TENS) [48], cognitive behavioural therapy, physical activity or behavioural health interventions [120], as well as cooling and compression techniques [125]. Those strategies were rated as low-quality evidence, except physical activity, behavioural activation and TENS, which had moderate quality evidence in the trauma and post-surgical context, respectively.

#### Others (surgical, alternative, psychological)

The remaining ten studies included seven RCTs [54, 55, 59, 61, 95, 113, 114] and three retrospective cohort study [60, 111, 112]. Four studies compared two types of surgery (inpatient or outpatient) with one another [59–61, 111]; one opioid use following Outpatient versus Inpatient Total Joint Arthroplasty [112]; three psychological interventions (Acceptance and Commitment Therapy, Mindfulness, Motivational interviewing with guided opioid tapering support) to information [95, 113]; and the last two evaluated the efficacy of acupuncture compared to usual care [54] or placebo [55]. All studies involved an orthopaedic surgical population except for one study on acupuncture that involved trauma patients. None of the interventions described in these studies [54, 55, 59–61, 95, 111, 112] showed a significant reductions in opioid use excepting one study evaluating the impact of an outpatient surgical intervention and two studies that assessed psychological strategies (mindfulness therapy and motivational interviewing combined with opioid tapering support). These studies demonstrated a statistically significant decrease in the proportion of new opioid persistent use [112], opioid use at 1 month [113] and an earlier return to previous opioid use [114] in elective orthopaedic surgery patients. A large proportion of these patients had psychological comorbidities and a history of preoperative opioid use particularly when psychological interventions were tested. We identified no guidelines recommendations on these intervention categories used as a single therapy.

### The effect of chronic opioid use prevention strategies on pain management

We analyzed study findings to determine if reductions in opioid use were associated with increased pain intensity. Near half of included studies (*n* = 30) assessed pain intensity concomitantly with strategies aimed at preventing chronic opioid use [53–55, 57–62, 64–68, 70, 71, 74–76, 78, 84, 90, 95, 96, 100, 106, 107, 110, 113, 114]. There was no significant difference between the groups that received a preventive strategy compared to the control groups for most of these studies, while seven indicated a significant decrease [57, 62, 65, 66, 71, 107, 113] and one a significant increase [78].

## Discussion

Our scoping review provides a comprehensive overview of the existing strategies to prevent long-term opioid use in patients who have undergone trauma or orthopeadic surgery while identifying future research avenues. More than 80% of studies and guidelines were published after 2017, reflecting the marked interest in countering the opioid crisis from the middle of the last decade. This concern is also highlighted by the fact that more than a third of the studies evaluated strategies to legislate or guide opioid prescriptions. Most of the studies were conducted in patients who had orthopaedic surgery and only a few were performed specifically in the trauma population. However, outcomes related to the different categories of strategies were comparable across these two types of study populations, even though elective orthopedic surgery patients were often using opioids preoperatively. Less than half of studies were high-quality evidence (i.e., RCTs). Retained studies evaluated system-based, pharmacological, educational, multimodal, surgical, alternative and psychological strategies. The most commonly used outcome measure was MED and a few studies documented the proportion of patients still using opioids at 3 months and beyond, which was considerably more important in those with risk factors. Data were mostly collected from patient records and clinical-administrative databases, but close to a third of studies did not provide information on the data source. Also, very few studies examined patient-reported outcomes and none measured the illicit use of opioids or opioid diversion that might be associated with a decrease in prescriptions [29, 31].

System-based strategies were regularly associated with long-term reductions in opioid use with mostly favourable results [78, 82, 83, 86, 102, 104, 105]. Such findings might indicate that hospital-based protocols and guidelines provide information on standards of practice and can be used as reminder mechanisms, enhancing the judicious prescription of opioids [135]. Legislations on prescribing limits were mainly effective with regard to the reduction of MED used by patients in the acute phase [85–88, 91, 93, 103, 104] but the effect was not sustained in the chronic phase in non-opioid naïve patients [87, 88, 91]. Prescribing limits enacted by several U.S. states have been associated with mitigated findings [136–138]. This may be caused by the fact that this strategy does not acknowledge the difference between minimally painful conditions and more painful conditions, and that it is not suitable for patients who were previously using opioids [139]. This more restrictive strategy may also lead patients to turn to the illicit market if pain remains a significant problem [31, 140]. For patients with risk factors for long-term opioid use, a more individualized approach to prescribing may be necessary as demonstrated by the positive results obtained in studies evaluating tailored tapering protocols [83, 84].

Regarding pharmacological strategies, only NSAIDs [71, 76] and beta blockers in specific orthopaedic surgical procedures limited prolonged opioid therapy. Ketamine has been the subject of several studies, but none of them were included in this review because opioid use was measured only in the short term (Supplemental Digital File 3: Excluded full texts). Interestingly, analgesics administered through nerve blocks and other drugs were mainly prescribed intraoperatively and in the immediate postoperative period, while NSAIDs [71, 76] and beta blockers [72] were used by patients for 6 weeks after surgery. Pregabalin, administered for approximately the same time period, did not lead to a decrease in MED in trauma patients. Nevertheless, this result was from a small RCT which was not powered to find a statistically significant difference in opioid use between groups [66]. Non-medical cannabis use also resulted in increased opioid consumption over a longer time period. However, despite its potential analgesic properties, the use of cannabis without medical supervision may indicate a propensity for substance abuse [141, 142]. Thus, extending medical prescriptions of co-analgesia beyond hospital discharge may be a potential solution to limit the long-term consumption of opioids. Such an approach will need to balance the risk-benefit of analgesics, such as NSAIDs which is contraindicated in patients with cardiovascular and renal diseases, and the risk of complications, which includes delayed bone union or non-union with more than 2 weeks of treatment [143–146].

Although fewer in number, studies on educational and multimodal preventive strategies also showed promising findings. Educational strategies included formal education to patients, with or without a follow-up, on how to use opioids and on the potential adverse events associated with prolonged therapy [5, 50, 53, 100, 106]. This indicates that some patients are responsive to advice provided by health care professionals on the risks associated with opioid use. The implementation of an educational assistive device to be used as a memory prompt about guidelines also helped health care professionals prescribe less opioids [52]. This concurs with the findings from a recent systematic review showing that interventions providing support during clinical decision can reduce low value practices [147]. With regard to multimodal strategies, those associated with positive outcomes were based on the concomitant use of several analgesics (e.g., acetaminophen, gabapentin, NSAID and opioid) [57, 68, 84, 101, 110], sometimes in combination with non-pharmacological strategies such as physiotherapy [58] and cryotherapy [56, 108] as well as the involvement of interdisciplinary teams [109]. In addition, the use of psychological strategies involving mindfulness [113] and motivational interviewing [114] have been shown to be beneficial in patients at risk for long-term opioid use following orthopaedic elective surgery. Such approaches were shown to improve pain management after musculoskeletal injuries [148, 149] and could, therefore, contribute to a decrease in opioid use. Conversely, although understudied, non-pharmacological strategies, such as the use of acupuncture were not conclusive [54, 55].

The evidence described in this review is largely similar to that of a recent systematic review on strategies to improve the judicious use of opioids in patients already on chronic therapy [34]. This review identified the following strategies as the most promising: clinical practice changes, such as a tool to improve opioid prescription practices, public campaigns, including the development of opioid prescribing guidelines, education for patients and health professionals, and collaborative work involving interprofessional and interdisciplinary teams [34]. However, although some strategies to prevent long-term opioid use after trauma and orthopaedic surgery were associated with favourable outcomes in terms of opioid use, the quality of evidence to support them remains low as highlighted in several recommendations from practice guidelines. This, except for recommendations on pharmacological strategies to use minimal opioids in trauma [47], multimodal pain management strategies [47] and NDSAIDs in orthopedic surgery [127, 132]. Several systematic reviews on opioid misuse in the context of chronic pain also concluded that there is a shortage of high-quality studies on strategies to promote the judicious use of opioids [150–152]. Many aspects of this complex issue will require further studies to enable the implementation of efficient and safe strategies in the health care setting. For example, even though the trauma population shares similarities with the surgical population, their care trajectories can lead to important gaps and setbacks in opioid weaning. Only a few hospitals are designated as trauma centers, so trauma patients may be sent to recover in regional hospitals while many may also necessitate rehabilitation before returning home and being monitored in their community [153]. Hence, strategies that target judicious opioid reduction in each phase of the trauma patient’s care trajectory, and communication mechanisms between health professionals involved in these different phases should be developed and evaluated in future studies. Furthermore, considering that the prescription of opioids has been identified as a precipitating factor in the illicit use of this drug and its derivatives, such as heroin [29, 30], it is important to develop strategies that take this risk into consideration. To this end, strategies that do not aim at stopping opioids at all costs but according to specific indicators, such as pain interference with activities, and that include patient follow-up, particularly those with risk factors for addiction, should be considered [140, 154].

In addition to reliable data on opioid use, future trials on the effectiveness of preventive strategies should focus on patient relevant outcomes such as pain, quality of life or daily function. Adverse events related to opioid use (e.g., intoxications, drowsiness, constipation, psychological distress), opioid diversion, illicit drug use as well as direct costs (e.g., health care service utilization, cost per quality-adjusted life-year) and indirect costs (e.g., lost in productivity) should also be measured. Likewise, the effectiveness of strategies in high-risk patients needs further confirmations considering that they use opioids in the long-term in a greater proportion, making them those who could benefit the most from preventive measures. Finally, subgroup analyses could help determine the role of biological sex and gender determinants, as well as socioeconomic status on the effect of preventive strategies [155, 156].

## Strengths and limits

This study presents a rigorous, comprehensive review of the evidence on strategies aimed at preventing chronic opioid use. Several trauma and surgery stakeholders from various disciplines (e.g., surgeons, physicians specialized in pain, psychologists, nurses, pharmacists and physiotherapists) and researchers specialized in trauma, orthopaedic surgery and/or mental health and addiction contributed to the analysis and interpretation of findings. These experts also identified research needs to decrease the knowledge gap regarding preventive strategies in order to determine their effectiveness and promote their implementation in clinical practice.

This study also has some limitations. First, for feasibility reasons, we restricted the review to studies and guidelines published since 2005. Hence, we may have missed research evidence published before this date. However, most studies on limiting opioid prescribing emerged after 2005, as illustrated by the fact that we only found studies or guidelines published since 2008 and beyond and only one item before 2010. Second, some types of preventive strategies, including alternative, have not been the subject of many large-scale studies, which limits the conclusions that can be drawn on their potential benefits. Third, significant findings on long-term opioid use are limited since the outcomes were sometimes measured no longer than 1 month after trauma or surgery. Nonetheless, we believe that data on medium-term use provide valuable information on opioid tapering trends extending after this period. Fourth, this review aimed to provide an overview of the research strategies to prevent chronic opioid use and the methodological quality of studies and guidelines was not assessed. Hence, although positive and significant results were identified for a few strategies, with some guidelines giving specific levels of evidence with regard to these strategies, findings must be interpreted with caution. Moreover, we do not know if reductions in prescribed opioid use for the studied strategies led patients to obtain this drug through non-legal means. In any case, findings highlight the need to conduct further studies to confirm the effectiveness and safety of the described preventive strategies. As well, to identify strategies to target for future research, we will need to determine those estimated to be the most feasible and useful by health care providers through a practice survey. This step will be included in a research program on the development and evaluation of strategies aimed at preventing long-term opioid use in high-risk trauma patients.

## Conclusion

Our scoping review gives an overview of the existing preventive strategies for chronic opioid use in patients who have undergone trauma and orthopaedic surgery. Some strategies, such as the implementation of individualized opioid tapering protocols, multimodal approaches, and educational strategies were promising. However, the low-quality evidence of these strategies clearly demonstrates that continued development and testing is needed to determine their preventive effect. In order to do so, future studies should target patients at high risk of chronic opioid use, evaluate patient-relevant and social outcomes, as well as measure opioid illicit use. More research on trauma patients who have specific care trajectories and on the potential risk of patients turning to illegitimate drug use is also required. Finally, special attention should be given to the feasibility and acceptability of the preventive strategies in complex trauma systems to facilitate their implementation in clinical practice.

## Supplementary Information

**Additional file 1.** Preferred Reporting Items for Systematic reviews and Meta-Analyses extension for Scoping Reviews (PRISMA-ScR) Checklist.

**Additional file 2.** Search Strategy in Medline.

**Additional file 3.** Excluded full texts.

**Additional file 4.** Risk factor for chronic opioid use in included studies by type of strategies.

## Data Availability

All data generated or analysed during the current study are included in this article and its supplementary information files.
