# Supplementary material for: Strategies aimed at preventing long-term opioid use in trauma and orthopaedic surgery: a scoping review
Source: BMC Musculoskelet Disord. 2022 Mar 11;23:238. doi: 10.1186/s12891-022-05044-y (PMC8917706; doi:10.1186/s12891-022-05044-y)
Supplement: Supplementary file 2 — Additional file 2. Search Strategy in Medline. [file 12891_2022_5044_MOESM2_ESM.docx]

**Supplemental Digital File 2. Search Strategy in Medline**

| **Concepts** | **Ovid search strategy keywords** | **Research** |
| --- | --- | --- |
| **Injuries and acute care surgeries** | exp "Wounds and Injuries"/ | #1 |
|  | (dislocation* or fracture* or injur* or traum* or "emergency surgery" OR "emergency surgeries").ab,kf,kw,sh,ti,tw.  ("Acute care surgery").ab,ti. OR ("Acute care surgeries").ab,ti. OR ("Ankle surgery").ab,ti. OR ("Ankle surgeries").ab,ti. OR ("Arthroplasty").ab,ti. OR ("Arthroplasties").ab,ti. OR ("Elbow surgery").ab,ti. OR ("Elbow surgeries").ab,ti. OR ("Hip surgery").ab,ti. OR ("Hip surgeries").ab,ti. OR ("Joint surgery").ab,ti. OR ("Joint reconstruction").ab,ti. OR ("Knee surgery").ab,ti. OR ("Knee surgeries").ab,ti. OR ("Orthopedics").ab,ti. OR ("Orthopaedics").ab,ti. OR ("Orthopedic procedure").ab,ti. OR ("Orthopedic procedures").ab,ti. OR ("Orthopaedic procedure").ab,ti. OR ("Orthopaedic procedures").ab,ti. OR ("Orthopedic surgery").ab,ti. OR ("Orthopaedic surgery").ab,ti. OR ("Orthopaedic surgeries").ab,ti. OR ("Orthopaedic surgeries").ab,ti. OR ("Shoulder surgery").ab,ti. OR ("Shoulder surgeries").ab,ti. OR ("Wrist surgery").ab,ti. OR ("Wrist surgeries").ab,ti.  ("Acute care surgery" OR "Acute care surgeries" OR "Ankle surgery" OR "Ankle surgeries" OR "Arthroplasty" OR "Arthroplasties" OR dislocation* OR "Elbow surgery" OR "Elbow surgeries" OR "emergency surgery" OR "emergency surgeries" OR fracture* OR "Hip surgery" OR "Hip surgeries" OR injur* OR "Joint replacement" OR "Joint surgery" OR "Joint reconstruction" OR "Knee surgery" OR "Knee surgeries" OR "Orthopedics" OR "Orthopaedics" OR "Orthopedic procedure" OR "Orthopedic procedures" OR "Orthopaedic procedure" OR "Orthopaedic procedures" OR "Orthopedic surgery" OR "Orthopaedic surgery" OR "Orthopaedic surgeries" OR "Orthopaedic surgeries" OR "Shoulder surgery" OR "Shoulder surgeries" OR traum* OR "Wrist surgery" OR "Wrist surgeries").ab,kf,kw,sh,ti,tw. | #2  #3 |
|  | 1 or 2 | #3 |
| **Opioids**  (Controlled vocabulary)  (Free text) | Alfentanil/ OR Buprenorphine, Naloxone Drug Combination/ OR Buprenorphine/ OR Morphine/ OR Fentanyl/ OR Hydrocodone/ OR Hydromorphone/ OR Levorphanol/ OR Meperidine/ OR Methadone/ OR Morphine Derivatives/ OR Oxycodone/ OR Oxymorphone/ OR Pentazocine/ OR Tramadol/ OR Narcotics/ OR exp Analgesics, Opioid/ OR exp Codeine/ OR Opioid-Related Disorders/ | #4 |
|  | (Alfentanil or Buprenorphine or Naloxone or Morphine or Fentanyl or Hydrocodone* or Hydromorphone or Levorphanol or Meperidine or Methadone or Morphine or Oxycodone or Oxymorphone or Pentazocine or Tramadol or Narcotics or Analgesic* or Opioid* or Codeine or opiate).ab,kf,kw,sh,ti,nm,tw. | #5 |
|  | 4 or 5 | #6 |
| **Preventive strategies**  (Controlled vocabulary)  (Free text) | "Acceptance and commitment therapy"/ OR Acetaminophen/ OR "Acupuncture Therapy"/ or Acupuncture/ OR Acupressure/ OR "Electroacupuncture"/ OR "Adaptation, Psychological"/ OR "Adrenergic alpha-2 Receptor Agonists"/ OR Clonidine/ OR "Analgesia"/ OR "Analgesics, Non-Narcotic"/ OR "Anesthetics, Local"/ OR "Anticonvulsants"/ OR "Antidepressive Agents"/ OR "Anti-Inflammatory Agents"/ OR "Biofeedback, Psychology"/ OR exp "Cognitive behavioral therapy"/ OR "Combined Modality Therapy"/ OR Counseling/ OR Cryotherapy/ OR "Decision Support Systems, Clinical"/ OR Exercise/ OR "Exercise Therapy"/ OR "Hyperthermia, Induced"/ OR Hypnosis/ OR "Immobilization"/ OR Massage/ OR Meditation/ OR "Mind-body therapies"/ or "Imagery (psychotherapy)"/ OR "Nerve Block"/ OR "Pain Clinics"/ OR Exp "Pain Management"/ OR "Organization and Administration"/ OR "Patient Education as Topic"/ OR "Patient Positioning"/ OR "Physical Therapy Modalities"/ OR "Prescription Drug Monitoring Programs"/ OR "Receptors, N-Methyl-D-Aspartate" OR Ketamine/ OR Relaxation/ OR "Relaxation Therapy"/ OR "Secondary Prevention"/ OR "Self Care"/ OR "Self-Management"/ OR "Sensory art therapies"/ or "Music therapy"/ OR "Transcutaneous Electric Nerve Stimulation"/ | #7 |
|  | ("acetaminophen").ab,ti. OR ("acupressure").ab,ti. OR ("acupuncture").ab,ti. OR ("adjuvant analgesics").ab,ti. OR ("adjuvant analgesic").ab,ti. OR ("analgesia").ab,ti. OR ("antidepressant").ab,ti. OR ("antidepressants").ab,ti. OR ("antiepileptic drug").ab,ti. OR ("antiepileptic drugs").ab,ti. OR ("anticonvulsant drug").ab,ti. OR ("anticonvulsant drugs").ab,ti OR ("clonidine").ab,ti. OR ("co-analgesics").ab,ti. OR ("co-analgesic").ab,ti. OR ("cognitive strategies").ab,ti. OR ("cognitive and emotional strategies").ab,ti. OR ("cryotherapy").ab,ti. OR ("discontinuation").ab,ti. OR ("distraction").ab,ti. OR ("emotional strategies").ab,ti. OR ("gabapentin").ab,ti. OR ("guided imagery").ab,ti. OR ("health system intervention").ab,ti. OR ("hypnosis relaxation").ab,ti. OR ("interdisciplinary pain management").ab,ti. OR ("ketamine").ab,ti. OR ("local analgesics").ab,ti. OR ("massage").ab,ti. OR ("meditation").ab,ti. OR ("multidisciplinary pain management").ab,ti. OR ("multimodal analgesia").ab,ti. OR ("nerve block").ab,ti. OR ("nerve stimulation").ab,ti. OR ("non narcotic analgesic").ab,ti. OR ("non-narcotic analgesic").ab,ti. OR ("nonnarcotic analgesic").ab,ti. OR ("non narcotic analgesics").ab,ti. OR ("non-narcotic analgesics").ab,ti. OR ("nonnarcotic analgesics").ab,ti. OR ("non opioid analgesic").ab,ti. OR ("non-opioid analgesic").ab,ti. OR ("nonopioid analgesic").ab,ti. OR ("non opioid analgesics").ab,ti. OR ("non-opioid analgesics").ab,ti. OR ("nonopioid analgesics").ab,ti. OR ("pain consultation service").ab,ti. OR ("pain medication policy").ab,ti. OR ("pain medication prescribing").ab,ti. OR ("pain medication prescribing policy").ab,ti. OR ("patient positioning").ab,ti. OR ("peer support").ab,ti. OR ("peer Support-Based Groups").ab,ti. OR ("policies and procedures").ab,ti. OR ("pregabalin").ab,ti. OR ("prescriber education").ab,ti. OR ("prescription limits").ab,ti. OR ("prevention").ab,ti. OR ("public education").ab,ti. OR ("reduction").ab,ti. OR ("relapse prevention").ab,ti. OR ("restrictive opioid prescription protocol").ab,ti. OR ("self-help").ab,ti. OR ("stage of change").ab,ti. OR ("superficial cold").ab,ti. OR ("superficial heat").ab,ti. OR ("support group").ab,ti. OR ("system strategies").ab,ti. OR ("taper").ab,ti. OR ("tapering").ab,ti. OR ("topical analgesics").ab,ti. OR ("transcutaneous electric nerve stimulation").ab,ti. OR ("transitional pain service").ab,ti. OR ("weaning").ab,ti. | #8 |
|  | Opioid* adj3 (reduction or Prevention or Weaning or Discontinuation or Tapering or management or manage).tw,sh,kw,kf,oa.  (Prevent* adj2 (((Continued or chronic or "long-term" or prolonged) adj2 ("use" or usage)) or misuse or overdose)).tw,sh,kw,kf,oa.  ((Psychosocial* or education* or cognitive or behavior* or Acceptance or commitment or Motivational or Enhancement or Contingency or Exercise or relaxation or physical or music or acupuncture or heat or acupressure) adj2 (therap* or strateg* or management* or program* treatment* or intervention*)).tw,sh,kw,kf,oa. | #9  #10  #11 |
|  | 7 OR 8 OR 9 OR 10 OR 11 | #12 |
| **Total** | 3 AND 6 AND 12 | #13 |
|  | animals/ | #14 |
|  | humans/ | #15 |
|  | 14 not 15 | #16 |
|  | 13 not 16 | #17 |
|  | (2005 or 2006 or 2007 or 2008 or 2009 or 2010 or 2011 or 2012 or 2013 or 2014 or 2015 or 2016 or 2017 or 2018 or 2019).yr. | #18 |
|  | 17 and 18 | #19 |
