# Supplementary material for: Strategies aimed at preventing long-term opioid use in trauma and orthopaedic surgery: a scoping review
Source: BMC Musculoskelet Disord. 2022 Mar 11;23:238. doi: 10.1186/s12891-022-05044-y (PMC8917706; doi:10.1186/s12891-022-05044-y)
Supplement: Supplementary file 3 — Additional file 3. Excluded full texts. [file 12891_2022_5044_MOESM3_ESM.docx]

**Supplemental Digital File 3 : Excluded full texts**

Abstact

1. Altintas NDI, S.; Alaybeyoʇlu, F.; Aksoy, M. Lorazepam may offer help when weaning patients from prolonged sedation with opioids in the intensive care unit. Intensive Care Medicine. 2013.

2. Balci AEÖ, M. O.; Çakmak, M. Treatment alternatives for traumatic rib fractures: Comparison of operative fixation and conservative approach. Interactive Cardiovascular and Thoracic Surgery 2013.

3. Boezaart APG, C. F.; Deen, J. T.; Zasimovich, Y.; Prieto, H.; Parvataneni, H. K. A comprehensive protocol for postoperative pain control in elective total joint arthroplasty drastically reduces opioid prescriptions: A review of prescribing practices and outcomes. Pain Medecine (United_States). 2013;20(3).

4. Bohl DDL, P.; Ahn, J.; Mayo, B.; Massel, D. H.; Narain, A. S.; Hijji, F. Y.; Long, W. W.; Modi, K.; Singh, K. Multimodal versus patient controlled analgesia following anterior cervical decompression and fusion procedures. Spine Journal. 2016;16(10).

5. Coleman CG, P.; Droege, M.; Philpott, C.; Hanseman, D.; Nomellini, V.; Droege, C. Prevention of chronic pain with the use of continuous infusion ketamine in acute trauma related pain. Journal of the American College of Clinical Pharmacy. 2018;1(2).

6. Eker HEC, O. Y.; Aribogan, A.; Arslan, G. The evaluation of peripheral nerve blocks with steroids in the management of acute and chronic neuropathic pain due to trauma. European Journal Of Pain. 2009;13.

7. Famiyeh IMF, A. D. The management of post-operative and post-trauma pain with opioids in a rehabilitation hospital. Archives of Physical Medicine and Rehabilitation 2014;95(19).

8. Gordin YW, L.; Kurihara, C.; Campos, N.; Bushey, D.; Griffith, S.; Liu, R.; Spevak, C. Spinal cord stimulators at walter reed national military medical center: Clinical characteristics of patients at 3-month follow-up

Journal of Pain. 2017;18(4).

9. Hassel FA, F.; Baud, J.; Ekkerlein, H.; Foulongne, E.; Francaviglia, N.; Gumpert, R.; Huet, H.; Kunsky, A.; Maestretti, G.; Noriega, D.; Ould Slimane, M.; Perot, G.; Plascencia, M.; Queinnec, S.; Renaud, C.; Sabatier, P.; Theumann, N. A prospective multicentric observational study on the use of intravertebral implants for traumatic vertebral compression fracture treatment-12 months follow-up results. European Spine Journal. 2014;23(11).

10. Heard KH, J.; Kim, H. Long-term opioid use among patients taking opioids and treated for a minor painful condition in the ED. Academic Emergency Medicine 2015;22(5).

11. Inacio MCSH, C.; Roughead, E. E.; Pratt, N. L.; Graves, S. E. Risk factors for persistent and new chronic opioid use in patients undergoing total hip arthroplasty. Pharmacoepidemiology and Drug Safety 2016;25.

12. Kong JC, D.; Jiang, A.; Hiscock, R.; Keon-Cohen, Z. Preoperative fasting: Assessing compliance with guidelines and patients understanding at a tertiary referral centre. Anaesthesia and Intensive Care. 2019;47(1).

13. Kuntz JLJ, E. S.; Petrik, A. F.; Yang, X.; Smith, D. H. Identifying patients for clinical trials by risk stratification: The case of an intervention to reduce post-operative opioid use in total joint replacement. Pharmacoepidemiology and Drug Safety 2015;24.

14. Livingston CC, N.; Anderson, H. The impact of prescription nsaids on opioid use in opioid-naïve patients following orthopedic surgery. Journal of Managed Care and Specialty Pharmacy. 2019;25.

15. Muir JB, S. L. Effects on patient lifestyle and quality of life: two-year outcomes of three lumbar total disc replacement systems from the activL® multicenter randomized controlled IDE clinical trial. Spine Journal. 2015;15(10).

16. Muller CW, A. R.; Blevins Peratikos, M. B.; Morris, L. C.; Stringer, E. A., States) PMU. The association of preoperative opioid tapering with postoperative opioid use and health care utilization. Pain medecine. 2018;20(3).

17. Mullins GB, P.; MacDougall, P.; Milne, A. Opioid prescribing for hip and knee arthroplasty using provincial monitoring program data. Canadian Journal of Anesthesia. 2018;65(1).

18. Oursbourn TO, T. IV acetaminophen use in patients who underwent hip of knew surgery: A systematic chart review. Regional Anesthesia and Pain Medicine 2012;37(6).

19. Pagnano MW. Intra-articular infusion with bupivacaine decreased pain and opioid consumption after total knee arthroplasty. Journal of Bone & Joint Surgery American 2013;95(10).

20. Pramesh CSJ, S.; Ranganathan, P.; Patil, V.; Karimundackal, G.; Agarwal, V. Phase ii double-blind randomized trial comparing posterolateral thoracotomy versus nerve sparing thoracotomy for lung surgery (POTNEST)-impact of preservation of the neurovascular bundle during thoracotomy on post-operative pain. Journal of thoracic oncology. 2013;8.

21. Pruitt LCS, D. S.; Bucher, B. T.; Skarda, D. E. Implementation of a Continuous Quality Improvement Project to Reduce Postoperative Opioid Prescribing Across Surgical Specialties. Journal of the American College of Surgeons. 2019;229(4).

22. Sabesan VJS, R.; Petersen-Fitts, G.; Bou-Akl, T. A prospective randomized trial to identify the optimal postoperative pain management in shoulder arthroplasty: liposomal bupivacaine vs. continuous peripheral nerve block. Journal of shoulder and elbow surgery 2017;26(5).

23. Sculco TP. Local infiltration analgesia reduced postoperative morphine consumption and provided better pain relief than intrathecal morphine after total knee arthroplasty. Journal of Bone & Joint Surgery American. 2016;94(16).

24. Starr JB, T.; Backonja, M.; Rozel, I. Antidepressant Use in Patients with a History of Depression is Associated with Reduced Opioid Use after Total Knee Arthroplasty. Journal of Pain 2019;20(4).

25. Szeverenyi CK, Z.; Elkins, G.; Csernatony, Z.; Varga, K.; Johnson, A. Psychosocial interventions as adjuncts to orthopedic surgery: A systematic review and meta-analysis. Global Advances in Health and Medicine. 2018;7.

26. Thompson MF, C.; Coffey, R.; Bailey, J. K.; Cochran, A.; Murphy, C. V. Opioid prescription patterns for burn injury: Before and after changes in state law. Journal of Burn Care and Research 2019;40.

27. Traylor EG, P.; Pollard, E. Role of Physical Therapy in Reducing Postoperative Musculoskeletal Pain In-Lieu of Opioid Pain Medications. Archives of Physical Medicine and Rehabilitation 2019;100(10).

28. Wandner LK, C.; Luong, Q.; Griffith, S.; Liu, R. Spinal cord stimulators at walter reed national military medical center: Opioid prescription practices at 6-month follow-up. Journal of Pain. 2018;19(3).

29. Zaffagnini S. Gabapentin did not reduce morphine consumption, pain, or opioid-related side effects in total knee arthroplasty. Journal of Bone & Joint Surgery American 2013;95(22).

30. Young JC, Dasgupta, N., Pate, V., Sturmer, T., Chidgey, B. A., & Funk, M. J. Estimating the impact of prescribing limits on prolonged opioid use following surgery. Pharmacoepidemiol Drug Saf. 2020;29:157.

31. Guidry C, Dema, B., Allen, C., & Stewart, D. Impact of state legislation on opioid prescribing practices of orthopedic surgeons. Journal of the American College of Clinical Pharmacy. 2020;3(8):1643.

Item not available

32. Erlendson M. Femoral and lateral femoral cutaneous nerve block in elderly hip fracture - A pilot study USA: Yale 2019.

Protocol

33. Actrn. Perioperative use of steroids in knee arthroplasty trial. <http://wwwwhoint/trialsearch/Trial2aspx?TrialID=ACTRN12619001090101>. 2019.

34. Brown R, Deyo B, Riley C, Quanbeck A, Glass JE, Turpin R, Hetzel S, Nicholas C, Cruz M, Agarwal S. Screening in Trauma for Opioid Misuse Prevention (STOMP): study protocol for the development of an opioid risk screening tool for victims of injury. Addiction Science & Clinical Practice. 2017;12(1):04.

35. Bugada D, De Gregori M, Compagnone C, Muscoli C, Raimondi F, Bettinelli S, Avanzini MA, Cobianchi L, Peloso A, Baciarello M, et al. Continuous wound infusion of local anesthetic and steroid after major abdominal surgery: study protocol for a randomized controlled trial. Trials. 2015;16:357.

36. Chi CI. Analgesic effects and early knee function of different analgesia in fast-track total knee arthroplasty without tourniquet. <http://wwwwhoint/trialsearch/Trial2aspx?TrialID=ChiCTR-INR-17012549>. 2017.

37. Chi CI. Single subsartorious femoral triangle block and adductor canal block for analgesia after primary total knee arthroplasty-a randamized, double-blind study. <http://wwwwhoint/trialsearch/Trial2aspx?TrialID=ChiCTR-INR-17012716>. 2017.

38. Chi CI. The early analgesia effects and functional rehabilitation of different analgesia in fast-track total knee arthroplasty without tourniquet. <http://wwwwhoint/trialsearch/Trial2aspx?TrialID=ChiCTR-INR-17012448>. 2017.

39. Chi CI. The safety and efficacy of intra-articular and peri-articular injection in total knee arthroplasty. <http://wwwwhoint/trialsearch/Trial2aspx?TrialID=ChiCTR-INR-16010099>. 2016.

40. Chi CI. clinical study of the therapeutic effect of microwave and ultrasonic on scar pain. <http://wwwwhoint/trialsearch/Trial2aspx?TrialID=ChiCTR-IOR-15005960>. 2015.

41. Chi CT. Effect of Ultrasound Guided Adductor Canal Block on Intraoperative hemodynamics, Postoperative Analgesia and Motor Function Recovery in Patients After Total Knee Arthroplasty. <http://wwwwhoint/trialsearch/Trial2aspx?TrialID=ChiCTR-TRC-14004669>. 2014.

42. Ctri. A COMPARATIVE STUDY OF Preoperative ANALGESIC EFFICACY OF ORAL GABAPENTIN 600mg VERSUS ORAL PREGABALIN 75mg FOR ORTHOPEDIC SURGERY UNDER SPINAL ANAESTHESIA. <http://wwwwhoint/trialsearch/Trial2aspx?TrialID=CTRI/2019/01/017050>. 2019.

43. Ctri. A study to determine the comparative efficacy of three different doses of morphine in the control of pain during knee surgery. <http://wwwwhoint/trialsearch/Trial2aspx?TrialID=CTRI/2015/02/005490>. 2015.

44. Ctri. Comparison of different nerve blocks for total knee replacement surgery. <http://wwwwhoint/trialsearch/Trial2aspx?TrialID=CTRI/2015/08/006126>. 2015.

45. Euctr NO. Pain after operations for radius fractures. <http://wwwwhoint/trialsearch/Trial2aspx?TrialID=EUCTR2016-000684-16-NO>. 2016.

46. Euctr DK. The effect of anaesthesia of the phrenic nerve to prevent shoulder pain after major lung surgery. <http://wwwwhoint/trialsearch/Trial2aspx?TrialID=EUCTR2012-002844-25-DK>. 2012.

47. Isrctn. Tapering opioids for trauma patients to reduce long-term opioid use. <http://wwwwhoint/trialsearch/Trial2aspx?TrialID=ISRCTN40263056>. 2018.

48. Mohammad HR, Trivella M, Hamilton TW, Strickland L, Murray D, Pandit H. Perioperative adjuvant corticosteroids for post-operative analgesia in elective knee surgery - A systematic review. Systematic Reviews. 2017;6.

49. Nct. Role of Pregabalin in Treatment of Post-Op Pain in Fracture Patients. <https://clinicaltrialsgov/show/NCT00583869>. 2007.

50. Nct. Use of Local Infiltration Analgesia Following Total Hip Arthroplasty. <https://clinicaltrialsgov/show/NCT01344395>. 2010.

51. Nct. Study to Test the Efficacy of Periarticular Levo Bupivicaine Injection Following Primary Hip Arthroplasty. <https://clinicaltrialsgov/show/NCT01106001>. 2010.

52. Nct. Oral Clonidine & Gabapentin: improving Recovery and Pain Management After Outpatient With Major Orthopedic Surgery. <https://clinicaltrialsgov/show/NCT01112878>. 2010.

53. Nct. Transversalis Fascial Plane Nerve Block in Iliac Crest Bone Graft. <https://clinicaltrialsgov/show/NCT01133730>. 2010.

54. Nct. Evaluation of Electrical Nerve Stimulation (TENS) Therapy for Pain Relief Following Total Knee Arthroplasty (TKA). <https://clinicaltrialsgov/show/NCT01641471>. 2012.

55. Nct. Motor Sparing Block vs. Peri-Articular Catheters. <https://clinicaltrialsgov/show/NCT01503528>. 2012.

56. Nct. Ultrasound Guided Fascia Iliaca Block for Pain Control After Elective Hip Replacement Surgery. <https://clinicaltrialsgov/show/NCT02108847>. 2014.

57. Nct. Continuous Femoral Nerve Block Versus Local-wound Infiltration Analgesia For Patients Receiving Total Knee Arthroplasty --- A Randomized Controlled Trial. <https://clinicaltrialsgov/show/NCT02284620>. 2014.

58. Nct. Operative Treatment of Unstable Thoracic Cage Injuries and Chest Wall Deformity in Trauma. <https://clinicaltrialsgov/show/NCT02132416>. 2014.

59. Nct. Effect of a Multimodal Pain Regimen on Pain Control, Patient Satisfaction and Narcotic Use in Orthopaedic Trauma Patients. <https://clinicaltrialsgov/show/NCT02160301>. 2014.

60. Nct. Single Shot Fascia Iliaca Block vs Femoral Nerve Block for Analgesia for Surgical Fixation of Hip Fractures. <https://clinicaltrialsgov/show/NCT02330302>. 2015.

61. Nct. Primary Care Intervention to Reduce Prescription Opioid Overdoses. <https://clinicaltrialsgov/show/NCT02464410>. 2015.

62. Nct. Effect of Gabapentin Enacarbil on Opioid Consumption and Pain Scores. <https://clinicaltrialsgov/show/NCT02840240>. 2016.

63. Nct. Anesthesia for Pain After Ankle Fracture Surgery. <https://clinicaltrialsgov/show/NCT02950558>. 2016.

64. Nct. Remote Controlled Analgesia on Patient Experience. <https://clinicaltrialsgov/show/NCT02720965>. 2016.

65. Nct. Does Continuous Adductor Canal Nerve Block Improve the Quality of Recovery for Outpatient Total Knee Arthroplasty Patients? <https://clinicaltrialsgov/show/NCT03038425>. 2017.

66. Nct. Altering The Transition From Acute to Chronic Pain (ATTAC-Pain). <https://clinicaltrialsgov/show/NCT03315533>. 2017.

67. Nct. Postoperative Analgesia After Elective Hip Surgery - Effect of Obturator Nerve Blockade. <https://clinicaltrialsgov/show/NCT03064165>. 2017.

68. Nct. Lidocaine 5% Patch (Lidoderm) for the Perioperative Prevention of Acute and Chronic Chest Pain Following Robotic Valve Surgery. <https://clinicaltrialsgov/show/NCT03120351>. 2017.

69. Nct. Pain Management of ACL Reconstruction. <https://clinicaltrialsgov/show/NCT03365908>. 2017.

70. Nct. Effect of Duloxetine on Opioid Use After Total Knee Arthroplasty. <https://clinicaltrialsgov/show/NCT03271151>. 2017.

71. Nct. Minimal Opioid Use After Total Hip Replacement (THR). <https://clinicaltrialsgov/show/NCT03090152>. 2017.

72. Nct. Buprenorphine Transdermal Patches in Arthroscopic Rotator Cuff Repair. <https://clinicaltrialsgov/show/NCT03380533>. 2017.

73. Nct. Peripheral Nerve Block Compared to Intra-articular Injection for TAA Post-operative Pain. <https://clinicaltrialsgov/show/NCT03674905>. 2018.

74. Nct. Comparative Evaluation of Lumbar Plexus and Suprainguinal Fascia Iliaca Compartment Blocks. <https://clinicaltrialsgov/show/NCT03746951>. 2018.

75. Nct. Analgesic Efficacy of US-Guided Interscalene Block Versus Supraclavicular Block for Ambulatory Arthroscopic Rotator Cuff Repair. <https://clinicaltrialsgov/show/NCT03743974>. 2018.

76. Nct. Analgesic Effect of TAP Block After Laparoscopic Cholecystectomy. <https://clinicaltrialsgov/show/NCT03391531>. 2018.

77. Nct. Ultrasound-Guided Percutaneous Peripheral Nerve Stimulation: a Department of Defense Funded Multicenter Study. <https://clinicaltrialsgov/show/NCT03481725>. 2018.

78. Nct. Intrathecal Morphine and Local Infiltration Analgesia in Total Knee Arthroplasty. <https://clinicaltrialsgov/show/NCT03434951>. 2018.

79. Nct. Effect of NSAID Use on Pain and Opioid Consumption Following Distal Radius Fracture. <https://clinicaltrialsgov/show/NCT03749616>. 2018.

80. Nct. Reducing Opioid Use for Chronic Pain Patients Following Surgery. <https://clinicaltrialsgov/show/NCT03675386>. 2018.

81. Nct. Multimodal Analgesia With Acetaminophen vs. Narcotics Alone After Hip Arthroscopy. <https://clinicaltrialsgov/show/NCT03510910>. 2018.

82. Nct. MAST Trial: multi-modal Analgesic Strategies in Trauma. <https://clinicaltrialsgov/show/NCT03472469>. 2018.

83. Nct. Relieving Acute Pain From Rib Fractures. <https://clinicaltrialsgov/show/NCT03426137>. 2018.

84. Nct. Multimodal Analgesia in Shoulder Arthroplasty. <https://clinicaltrialsgov/show/NCT03586934>. 2018.

85. Nct. Does Altering Narcotic Prescription Methods Affect Opioid Distribution Following Select Upper Extremity Surgeries? <https://clinicaltrialsgov/show/NCT03570320>. 2018.

86. Nct. Virtual Reality Analgesia In Trauma Rehab. <https://clinicaltrialsgov/show/NCT03894592>. 2019.

87. Nct. Liposomal Bupivacaine vs Peripheral Nerve Block. <https://clinicaltrialsgov/show/NCT03922620>. 2019.

88. Nct. SMS Education for Post-operative Pain. <https://clinicaltrialsgov/show/NCT04039191>. 2019.

89. Nct. Ultrasound-Guided Erector Spinae Plane Block Following Upper Extremity Surgery. <https://clinicaltrialsgov/show/NCT04083274>. 2019.

90. Nct. Ultrasound-Guided Erector Spinae Plane Block or Interscalen Brachial Plexus Block Following Arthroscopic Shoulder Surgery. <https://clinicaltrialsgov/show/NCT04083287>. 2019.

91. Nct. Effect of LIA on Postoperative Pain Following ACL Reconstruction. <https://clinicaltrialsgov/show/NCT03873077>. 2019.

92. Nct. Post-operative Exparel Study Following Rotator Cuff Repair. <https://clinicaltrialsgov/show/NCT04047745>. 2019.

93. Nct. Opioid Use Post-Discharge After Ambulatory Distal Arm Surgery. <https://clinicaltrialsgov/show/NCT04044820>. 2019.

94. Nct. Reduction of Opioid Dose Using Conditioning & Open-Label Placebo (COLP) in Spinal Cord Injury Patients. <https://clinicaltrialsgov/show/NCT03906721>. 2019.

95. Nct. Battlefield Acupuncture Following Shoulder Surgery. <https://clinicaltrialsgov/show/NCT04094246>. 2019.

96. Nct. The Effect of Prolonged Multimodal Analgesic Regimen on Post Hospital Discharge Opioid Use and Pain Control After Primary Total Knee Arthroplasty. <https://clinicaltrialsgov/show/NCT04003350>. 2019.

97. Nct. Hip Arthroscopy Postoperative Opioid Demands. <https://clinicaltrialsgov/show/NCT04094701>. 2019.

98. Omaki E, Castillo R, Eden K, Davis S, McDonald E, Murtaza U, Gielen A, My Healthy Choices Decision Aid Study T. Using m-health tools to reduce the misuse of opioid pain relievers. Injury Prevention. 2019;25(4):334-9.

99. Smits AJ, Deunk J, Stadhouder A, Altena MC, Kempen DHR, Bloemers FW. Is postoperative bracing after pedicle screw fixation of spine fractures necessary? Study protocol of the ORNOT study: a randomised controlled multicentre trial. BMJ open. 2018;8(1):e019596.

100. Stulberg JJ, Schafer WLA, Shallcross ML, Lambert BL, Huang RP, Holl JL, Bilimoria KY, Johnson JK. Evaluating the implementation and effectiveness of a multi-component intervention to reduce post-surgical opioid prescribing: study protocol of a mixed-methods design. Bmj Open. 2019;9(6).

101. Tctr. The Efficacy of Popliteal plexus Block for Total Knee Arthroplasty: a Randomized Controlled Trial. <http://wwwwhoint/trialsearch/Trial2aspx?TrialID=TCTR20180206002>. 2018.

102. Wijffels MME, Prins JTH, Polinder S, Blokhuis TJ, De Loos ER, Den Boer RH, Flikweert ER, Pull Ter Gunne AF, Ringburg AN, Spanjersberg WR, et al. Early fixation versus conservative therapy of multiple, simple rib fractures (FixCon): protocol for a multicenter randomized controlled trial. World journal of emergency surgery. 2019;14:38‐.

103. Zhuang Q, Bian Y, Wang W, Jiang J, Feng B, Sun T, Lin J, Zhang M, Yan S, Shen B, et al. Efficacy and safety of Postoperative Intravenous Parecoxib sodium Followed by ORal CElecoxib (PIPFORCE) post-total knee arthroplasty in patients with osteoarthritis: a study protocol for a multicentre, double-blind, parallel-group trial. BMJ open. 2016;6(9):e011732.

Wrong outcomes

104. Adam F, Chauvin M, Du Manoir B, Langlois M, Sessler DI, Fletcher D. Small-dose ketamine infusion improves postoperative analgesia and rehabilitation after total knee arthroplasty. Anesth Analg. 2005;100(2):475-80.

105. Aggarwal AK, Shashikanth VS, Marwaha N. Platelet-rich plasma prevents blood loss and pain and enhances early functional outcome after total knee arthroplasty: a prospective randomised controlled study. Int Orthop. 2014;38(2):387-95.

106. Albrecht EM, D.; Chan, V.; Gandhi, R.; Koshkin, A.; Chin, K. J.; Robinson, S.; Frascarolo, P.; Brull, R. Single-shot or continuous infusion femoral nerve blockade for total knee arthroplasty? A randomized, placebo-controlled, double-blind trial. Regional anesthesia and pain medicine. 2012;37(6).

107. Ali A, Sundberg M, Hansson U, Malmvik J, Flivik G. Doubtful effect of continuous intraarticular analgesia after total knee arthroplasty: a randomized double-blind study of 200 patients. Acta Orthop. 2015;86(3):373-7.

108. Alter TH, Ilyas AM. A Prospective Randomized Study Analyzing Preoperative Opioid Counseling in Pain Management After Carpal Tunnel Release Surgery. J Hand Surg Am. 2017;42(10):810-5.

109. Andersen L, Husted H, Kristensen BB, Otte KS, Gaarn-Larsen L, Kehlet H. Analgesic efficacy of subcutaneous local anaesthetic wound infiltration in bilateral knee arthroplasty: a randomised, placebo-controlled, double-blind trial. Acta Anaesthesiol Scand. 2010;54(5):543-8.

110. Andersen K, Nikolajsen L, Daugaard H, Andersen K, Haraldsted V, Søballe K. Local infiltration analgesia is not improved by postoperative intra-articular bolus injections for pain after total hip arthroplasty. Acta Orthop. 2015;86(6):647-53.

111. Andersen L, Husted H, Kristensen BB, Otte KS, Gaarn-Larsen L, Kehlet H. Analgesic efficacy of intracapsular and intra-articular local anaesthesia for knee arthroplasty. Anaesthesia. 2010;65(9):904-12.

112. Andrade-Silva FB, Rocha JP, Carvalho A, Kojima KE, Silva JS. Influence of postoperative immobilization on pain control of patients with distal radius fracture treated with volar locked plating: A prospective, randomized clinical trial. Injury. 2019;50(2):386-91.

113. Aras EL, Bunger C, Hansen ES, Søgaard R. Cost-Effectiveness of Surgical Versus Conservative Treatment for Thoracolumbar Burst Fractures. Spine (Phila Pa 1976). 2016;41(4):337-43.

114. Ashik MS-L, C.; Jin, Y. S.; Hong, T. M.; Nung, L. N. Comparison of the different modalities of post operative analgesia in unilateral total knee arthroplasty patients. Journal of Orthopaedics. 2010;7(1).

115. Baird J, Faul M, Green TC, Howland J, Adams CA, Jr., Hodne MJ, Bohlen N, Mello MJ. Evaluation of a Safer Opioid Prescribing Protocol (SOPP) for Patients Being Discharged From a Trauma Service. J Trauma Nurs. 2019;26(3):113-20.

116. Barrons RW, Woods JA. Low-Dose Naloxone for Prophylaxis of Postoperative Nausea and Vomiting: A Systematic Review and Meta-analysis. Pharmacotherapy. 2017;37(5):546-54.

117. Beaupre LA, Johnston DB, Dieleman S, Tsui B. Impact of a preemptive multimodal analgesia plus femoral nerve blockade protocol on rehabilitation, hospital length of stay, and postoperative analgesia after primary total knee arthroplasty: a controlled clinical pilot study. ScientificWorldJournal. 2012;2012:273821.

118. Beaussier M, Aissou M. [Continuous wound infiltration or intra-articular infusion for postoperative analgesia]. Ann Fr Anesth Reanim. 2009;28(3):e153-62.

119. Becchi C, Al Malyan M, Coppini R, Campolo M, Magherini M, Boncinelli S. Opioid-free analgesia by continuous psoas compartment block after total hip arthroplasty. A randomized study. Eur J Anaesthesiol. 2008;25(5):418-23.

120. Bingham AEF, R.; Horn, J. L.; Abrahams, M. S. Continuous Peripheral Nerve Block Compared With Single-Injection Peripheral Nerve Block A Systematic Review and Meta-Analysis of Randomized Controlled Trials. Regional Anesthesia and Pain Medicine. 2012;37(6):583-94.

121. Bjørnholdt KT, Jensen JM, Bendtsen TF, Søballe K, Nikolajsen L. Local infiltration analgesia versus continuous interscalene brachial plexus block for shoulder replacement pain: a randomized clinical trial. Eur J Orthop Surg Traumatol. 2015;25(8):1245-52.

122. Bugada D, Allegri M, Gemma M, Ambrosoli AL, Gazzerro G, Chiumiento F, Dongu D, Nobili F, Fanelli A, Ferrua P, et al. Effects of anaesthesia and analgesia on long-term outcome after total knee replacement: A prospective, observational, multicentre study. European Journal of Anaesthesiology. 2017;34(10):665-72.

123. Bunketorp L, Lindh M, Carlsson J, Stener-Victorin E. The effectiveness of a supervised physical training model tailored to the individual needs of patients with whiplash-associated disorders--a randomized controlled trial. Clin Rehabil. 2006;20(3):201-17.

124. Busch CA, Whitehouse MR, Shore BJ, MacDonald SJ, McCalden RW, Bourne RB. The efficacy of periarticular multimodal drug infiltration in total hip arthroplasty. Clin Orthop Relat Res. 2010;468(8):2152-9.

125. Carli F, Clemente A, Asenjo JF, Kim DJ, Mistraletti G, Gomarasca M, Morabito A, Tanzer M. Analgesia and functional outcome after total knee arthroplasty: periarticular infiltration vs continuous femoral nerve block. Br J Anaesth. 2010;105(2):185-95.

126. Cerfolio RJ, Bryant AS, Maniscalco LM. A nondivided intercostal muscle flap further reduces pain of thoracotomy: a prospective randomized trial. Ann Thorac Surg. 2008;85(6):1901-6; discussion 6-7.

127. Chalmers PNS, D.; Fingerman, M. E.; Keener, J. D.; Chamberlain, A. Continuous interscalene brachial plexus blockade is associated with reduced length of stay after shoulder arthroplasty. Orthopaedics & Traumatology-Surgery & Research 2017;103(6):847-52.

128. Chau JY, Chan WL, Woo SB, Cheng SC, Wong TM, Wong TK, Yen CH, Wong K, Wong WC. Hyaluronic acid instillation following arthroscopic anterior cruciate ligament reconstruction: a double-blinded, randomised controlled study. J Orthop Surg (Hong Kong). 2012;20(2):162-5.

129. Cheng BLY, So EHK, Hui GKM, Yung BPK, Tsui ASK, Wang OKF, Poon MWY, Chan ACM, Wong SHS, Li W, et al. Pre-operative intravenous steroid improves pain and joint mobility after total knee arthroplasty in Chinese population: a double-blind randomized controlled trial. Eur J Orthop Surg Traumatol. 2019;29(7):1473-9.

130. Chin KK, Carroll I, Desai K, Asch S, Seto T, McDonald KM, Curtin C, Hernandez-Boussard T. Integrating Adjuvant Analgesics into Perioperative Pain Practice: Results from an Academic Medical Center. Pain Med. 2020;21(1):161-70.

131. Choi YS, Shim JK, Song JW, Kim JC, Yoo YC, Kwak YL. Combination of pregabalin and dexamethasone for postoperative pain and functional outcome in patients undergoing lumbar spinal surgery: a randomized placebo-controlled trial. Clin J Pain. 2013;29(1):9-14.

132. Chughtai M, Sodhi N, Jawad M, Newman JM, Khlopas A, Bhave A, Mont MA. Cryotherapy Treatment After Unicompartmental and Total Knee Arthroplasty: A Review. J Arthroplasty. 2017;32(12):3822-32.

133. Clarke H, Pagé GM, McCartney CJ, Huang A, Stratford P, Andrion J, Kennedy D, Awad IT, Gollish J, Kay J, et al. Pregabalin reduces postoperative opioid consumption and pain for 1 week after hospital discharge, but does not affect function at 6 weeks or 3 months after total hip arthroplasty. Br J Anaesth. 2015;115(6):903-11.

134. Clarke HA, Katz J, McCartney CJ, Stratford P, Kennedy D, Pagé MG, Awad IT, Gollish J, Kay J. Perioperative gabapentin reduces 24 h opioid consumption and improves in-hospital rehabilitation but not post-discharge outcomes after total knee arthroplasty with peripheral nerve block. Br J Anaesth. 2014;113(5):855-64.

135. Clarke HM, C.; Page, G.; Kennedy, D.; Stratford, P.; Awad, I.; Kay, J.; Gollish, J.; Katz, J. Pregabalin reduces 24 H opioid consumption and improves daily pain for one week after discharge, but not function at six weeks, three months or six months following total hip arthroplasty. Pain research and management. 2014;19(3).

136. Clarke H, Kay J, Mitsakakis N, Katz J. Acute pain after total hip arthroplasty does not predict the development of chronic postsurgical pain 6 months later. J Anesth. 2010;24(4):537-43.

137. Clarke H, Pereira S, Kennedy D, Andrion J, Mitsakakis N, Gollish J, Katz J, Kay J. Adding gabapentin to a multimodal regimen does not reduce acute pain, opioid consumption or chronic pain after total hip arthroplasty. Acta Anaesthesiol Scand. 2009;53(8):1073-83.

138. Cruz Eng H, Riazi S, Veillette C, Ami N, Niazi AU, Chin KJ, Chan VW, Perlas A. An Expedited Care Pathway with Ambulatory Brachial Plexus Analgesia Is a Cost-effective Alternative to Standard Inpatient Care after Complex Arthroscopic Elbow Surgery: A Randomized, Single-blinded Study. Anesthesiology. 2015;123(6):1256-66.

139. Danovich D, Greenstein J, Chacko J, Hahn B, Ardolic B, Ilyaguyev B, Berwald N. Effect of New York State Electronic Prescribing Mandate on Opioid Prescribing Patterns. J Emerg Med. 2019;57(2):156-61.

140. De la Fuente C, Peña y Lillo R, Carreño G, Marambio H. Prospective randomized clinical trial of aggressive rehabilitation after acute Achilles tendon ruptures repaired with Dresden technique. Foot (Edinb). 2016;26:15-22.

141. Deiter J. Implementing a practice change: Improving pain and reducing narcotic utilization by evaluating the analgesic efficacy of administering an adductor canal block to patients undergoing a total knee arthroplasty. Dissertation Abstracts International: Section B: The Sciences and Engineering 2019. 2019;80(6-B(E)).

142. Dorr LD, Raya J, Long WT, Boutary M, Sirianni LE. Multimodal analgesia without parenteral narcotics for total knee arthroplasty. J Arthroplasty. 2008;23(4):502-8.

143. Du X, Gu J. The efficacy and safety of parecoxib for reducing pain and opioid consumption following total knee arthroplasty: A meta-analysis of randomized controlled trials. Int J Surg. 2018;59:67-74.

144. Dwyer CL, Soong M, Hunter A, Dashe J, Tolo E, Kasparyan NG. Prospective Evaluation of an Opioid Reduction Protocol in Hand Surgery. J Hand Surg Am. 2018;43(6):516-22.e1.

145. Ejaz A, Laursen AC, Kappel A, Laursen MB, Jakobsen T, Rasmussen S, Nielsen PT. Faster recovery without the use of a tourniquet in total knee arthroplasty. Acta Orthop. 2014;85(4):422-6.

146. El-Kerdawy H. Analgesic requirements for patients undergoing lower extremity orthopedic surgery--the effect of combined spinal and epidural magnesium. Middle East J Anaesthesiol. 2008;19(5):1013-25.

147. Ellis TA, 2nd, Hammoud H, Dela Merced P, Nooli NP, Ghoddoussi F, Kong J, Krishnan SH. Multimodal Clinical Pathway With Adductor Canal Block Decreases Hospital Length of Stay, Improves Pain Control, and Reduces Opioid Consumption in Total Knee Arthroplasty Patients: A Retrospective Review. J Arthroplasty. 2018;33(8):2440-8.

148. Essving P, Axelsson K, Otterborg L, Spännar H, Gupta A, Magnuson A, Lundin A. Minimally invasive surgery did not improve outcome compared to conventional surgery following unicompartmental knee arthroplasty using local infiltration analgesia: a randomized controlled trial with 40 patients. Acta Orthop. 2012;83(6):634-41.

149. Fei D, Ma LP, Yuan HP, Zhao DX. Comparison of femoral nerve block and fascia iliaca block for pain management in total hip arthroplasty: A meta-analysis. Int J Surg. 2017;46:11-3.

150. Friedstat JSL, J. M.; Sangji, N. F.; Bilodeau, M. C.; McSweeney, J. P.; Chang, K. M.; Goverman, J.; Schulz, J. T. A quality improvement project to tighten discharge opioid prescribing. Journal of Burn Care and Research. 2019;40.

151. Galos DK, Taormina DP, Crespo A, Ding DY, Sapienza A, Jain S, Tejwani NC. Does Brachial Plexus Blockade Result in Improved Pain Scores After Distal Radius Fracture Fixation? A Randomized Trial. Clin Orthop Relat Res. 2016;474(5):1247-54.

152. Grape S, Kirkham KR, Baeriswyl M, Albrecht E. The analgesic efficacy of sciatic nerve block in addition to femoral nerve block in patients undergoing total knee arthroplasty: a systematic review and meta-analysis. Anaesthesia. 2016;71(10):1198-209.

153. Gupta A, Favaios S, Perniola A, Magnuson A, Berggren L. A meta-analysis of the efficacy of wound catheters for post-operative pain management. Acta Anaesthesiol Scand. 2011;55(7):785-96.

154. Gwam CU, Mistry JB, Khlopas A, Chughtai M, Thomas M, Mont MA, Delanois RE. Does Addition of Multimodal Periarticular Analgesia to Adductor Canal Block Improve Lengths of Stay, Pain, Discharge Status, and Opioid Use After Total Knee Arthroplasty? J Arthroplasty. 2017;32(5):1470-3.

155. Hagedorn JCM, M.; Holihan, J.; Choo, A.; Achor, T.; Munz, J.; Gary, J. . **The opioid epidemic and patient satisfaction: A review of one institution's experience**. 29. 2018(6).

156. Hamilton TW, Strickland LH, Pandit HG. A Meta-Analysis on the Use of Gabapentinoids for the Treatment of Acute Postoperative Pain Following Total Knee Arthroplasty. J Bone Joint Surg Am. 2016;98(16):1340-50.

157. Han C, Kuang MJ, Ma JX, Ma XL. Is pregabalin effective and safe in total knee arthroplasty? A PRISMA-compliant meta-analysis of randomized-controlled trials. Medicine (Baltimore). 2017;96(26):e6947.

158. Hartrick CT, Tang YS, Siwek D, Murray R, Hunstad D, Smith G. The effect of initial local anesthetic dose with continuous interscalene analgesia on postoperative pain and diaphragmatic function in patients undergoing arthroscopic shoulder surgery: a double-blind, randomized controlled trial. BMC Anesthesiol. 2012;12:6.

159. Hussain N, Goldar G, Ragina N, Banfield L, Laffey JG, Abdallah FW. Suprascapular and Interscalene Nerve Block for Shoulder Surgery: A Systematic Review and Meta-analysis. Anesthesiology. 2017;127(6):998-1013.

160. Hyland SJ, Deliberato DG, Fada RA, Romanelli MJ, Collins CL, Wasielewski RC. Liposomal Bupivacaine Versus Standard Periarticular Injection in Total Knee Arthroplasty With Regional Anesthesia: A Prospective Randomized Controlled Trial. J Arthroplasty. 2019;34(3):488-94.

161. Ilfeld BM, Ball ST, Gabriel RA, Sztain JF, Monahan AM, Abramson WB, Khatibi B, Said ET, Parekh J, Grant SA, et al. A Feasibility Study of Percutaneous Peripheral Nerve Stimulation for the Treatment of Postoperative Pain Following Total Knee Arthroplasty. Neuromodulation. 2019;22(5):653-60.

162. Ingalls NK, Horton ZA, Bettendorf M, Frye I, Rodriguez C. Randomized, double-blind, placebo-controlled trial using lidocaine patch 5% in traumatic rib fractures. J Am Coll Surg. 2010;210(2):205-9.

163. Jain SK, Dar MY, Kumar S, Yadav A, Kearns SR. Role of anti-oxidant (vitamin-C) in post-operative pain relief in foot and ankle trauma surgery: A prospective randomized trial. Foot Ankle Surg. 2019;25(4):542-5.

164. Jones LM, Uribe AA, Coffey R, Puente EG, Abdel-Rasoul M, Murphy CV, Bergese SD. Pregabalin in the reduction of pain and opioid consumption after burn injuries: A preliminary, randomized, double-blind, placebo-controlled study. Medicine (Baltimore). 2019;98(18):e15343.

165. Joo JH, Park JW, Kim JS, Kim YH. Is intra-articular multimodal drug injection effective in pain management after total knee arthroplasty? A randomized, double-blinded, prospective study. J Arthroplasty. 2011;26(7):1095-9.

166. Kadic LB, M. C.; D. E. Waal Malefijt MC; Lako, S. J.; V. A. N. Egmond J; Driessen, J. J. Continuous femoral nerve block after total knee arthroplasty? Acta Anaesthesiologica Scandinavica. 2009;53(7).

167. Kazerooni R, Tran MH. Evaluation of Celecoxib Addition to Pain Protocol After Total Hip and Knee Arthroplasty Stratified by Opioid Tolerance. Clin J Pain. 2015;31(10):903-8.

168. King C, Curran J, Devanagondi S, Balach T, Conti Mica M. Targeted Intervention to Increase Awareness of Opioid Overprescribing Significantly Reduces Narcotic Prescribing Within an Academic Orthopaedic Practice. J Surg Educ. 2020;77(2):413-21.

169. Klement MR, Bullock WM, Nickel BT, Lampley AJ, Seyler TM, Green CL, Wellman SS, Bolognesi MP, Grant SA. Continuous adductor canal blockade facilitates increased home discharge and decreased opioid consumption after total knee arthroplasty. Knee. 2019;26(3):679-86.

170. Leach W, Reid J, Murphy F. Continuous passive motion following total knee replacement: a prospective randomized trial with follow-up to 1 year. Knee Surg Sports Traumatol Arthrosc. 2006;14(10):922-6.

171. Lemay CA, Lewis CG, Singh JA, Franklin PD. Receipt of Pain Management Information Preoperatively Is Associated With Improved Functional Gain After Elective Total Joint Arthroplasty. J Arthroplasty. 2017;32(6):1763-8.

172. Liu W, Cong R, Li X, Wu Y, Wu H. Reduced opioid consumption and improved early rehabilitation with local and intraarticular cocktail analgesic injection in total hip arthroplasty: a randomized controlled clinical trial. Pain Med. 2011;12(3):387-93.

173. López-Vidriero E, Olivé-Vilas R, López-Capapé D, Varela-Sende L, López-Vidriero R, Til-Pérez L. Efficacy and Tolerability of Progen, a Nutritional Supplement Based on Innovative Plasma Proteins, in ACL Reconstruction: A Multicenter Randomized Controlled Trial. Orthop J Sports Med. 2019;7(2):2325967119827237.

174. Louw A, Puentedura EJ, Reed J, Zimney K, Grimm D, Landers MR. A controlled clinical trial of preoperative pain neuroscience education for patients about to undergo total knee arthroplasty. Clin Rehabil. 2019;33(11):1722-31.

175. Macrinici GI, Murphy C, Christman L, Drescher M, Hughes B, Macrinici V, Diab G. Prospective, Double-Blind, Randomized Study to Evaluate Single-Injection Adductor Canal Nerve Block Versus Femoral Nerve Block: Postoperative Functional Outcomes After Total Knee Arthroplasty. Reg Anesth Pain Med. 2017;42(1):10-6.

176. Marion B, Klouche S, Deranlot J, Bauer T, Nourissat G, Hardy P. A Prospective Comparative Study of Arthroscopic Versus Mini-Open Latarjet Procedure With a Minimum 2-Year Follow-up. Arthroscopy. 2017;33(2):269-77.

177. Mayoral O, Salvat I, Martín MT, Martín S, Santiago J, Cotarelo J, Rodríguez C. Efficacy of myofascial trigger point dry needling in the prevention of pain after total knee arthroplasty: a randomized, double-blinded, placebo-controlled trial. Evid Based Complement Alternat Med. 2013;2013:694941.

178. McLaughlin DC, Cheah JW, Aleshi P, Zhang AL, Ma CB, Feeley BT. Multimodal analgesia decreases opioid consumption after shoulder arthroplasty: a prospective cohort study. J Shoulder Elbow Surg. 2018;27(4):686-91.

179. Memary E, Mirkheshti A, Dabbagh A, Taheri M, Khadempour A, Shirian S. The Effect of Perineural Administration of Dexmedetomidine on Narcotic Consumption and Pain Intensity in Patients Undergoing Femoral Shaft Fracture Surgery; A Randomized Single-Blind Clinical Trial. Chonnam Med J. 2017;53(2):127-32.

180. Merrill HM, Dean DM, Mottla JL, Neufeld SK, Cuttica DJ, Buchanan MM. Opioid Consumption Following Foot and Ankle Surgery. Foot Ankle Int. 2018;39(6):649-56.

181. Meunier A, Lisander B, Good L. Effects of celecoxib on blood loss, pain, and recovery of function after total knee replacement: a randomized placebo-controlled trial. Acta Orthop. 2007;78(5):661-7.

182. Mont MA, Beaver WB, Dysart SH, Barrington JW, Del Gaizo DJ. Local Infiltration Analgesia With Liposomal Bupivacaine Improves Pain Scores and Reduces Opioid Use After Total Knee Arthroplasty: Results of a Randomized Controlled Trial. J Arthroplasty. 2018;33(1):90-6.

183. Morrison RS, Flanagan S, Fischberg D, Cintron A, Siu AL. A novel interdisciplinary analgesic program reduces pain and improves function in older adults after orthopedic surgery. J Am Geriatr Soc. 2009;57(1):1-10.

184. Mullaji A, Kanna R, Shetty GM, Chavda V, Singh DP. Efficacy of periarticular injection of bupivacaine, fentanyl, and methylprednisolone in total knee arthroplasty:a prospective, randomized trial. J Arthroplasty. 2010;25(6):851-7.

185. Nakagawa S, Arai Y, Inoue H, Kan H, Hino M, Ichimaru S, Ikoma K, Fujiwara H, Amaya F, Sawa T, et al. Comparative Effects of Periarticular Multimodal Drug Injection and Single-Shot Femoral Nerve Block on Pain Following Total Knee Arthroplasty and Factors Influencing Their Effectiveness. Knee Surg Relat Res. 2016;28(3):233-8.

186. Osti L, Buono AD, Maffulli N. Pulsed electromagnetic fields after rotator cuff repair: a randomized, controlled study. Orthopedics. 2015;38(3):e223-8.

187. Page RS, Williams S, Selvaratnam A, Waring S, Conroy M, Thomson A, Beattie S, Ganeshalingam R, Gill SD. Protocol for a single-centre, parallel-arm, double-blind randomised trial evaluating the effects of tourniquet use in total knee arthroplasty on intra-operative and post-operative outcomes. BMC Musculoskelet Disord. 2018;19(1):435.

188. Panchamia JK, Amundson AW, Jacob AK, Sviggum HP, Nguyen NTV, Sanchez-Sotelo J, Sperling JW, Schroeder DR, Kopp SL, Johnson RL. A 3-arm randomized clinical trial comparing interscalene blockade techniques with local infiltration analgesia for total shoulder arthroplasty. J Shoulder Elbow Surg. 2019;28(10):e325-e38.

189. Peerbooms JCDW, G. S.; Colaris, J. W.; Bruijn, D. J.; Verhaar, J. A. N. No positive effect of autologous platelet gel after total knee arthroplasty: a double-blind randomized controlled trial: 102 patients with a 3-month follow-up. Acta orthopaedica 2009;80(5):557-62.

190. Peng YN, Sung FC, Huang ML, Lin CL, Kao CH. The use of intravenous magnesium sulfate on postoperative analgesia in orthopedic surgery: A systematic review of randomized controlled trials. Medicine (Baltimore). 2018;97(50):e13583.

191. Peng L, Ren L, Qin P, Chen J, Feng P, Lin H, Su M. Continuous Femoral Nerve Block versus Intravenous Patient Controlled Analgesia for Knee Mobility and Long-Term Pain in Patients Receiving Total Knee Replacement: A Randomized Controlled Trial. Evid Based Complement Alternat Med. 2014;2014:569107.

192. Previtali D, Di Laura Frattura G, Filardo G, Delcogliano M, Deabate L, Candrian C. Peri-operative steroids reduce pain, inflammatory response and hospitalisation length following knee arthroplasty without increased risk of acute complications: a meta-analysis. Knee Surg Sports Traumatol Arthrosc. 2019.

193. Rainville EC, Asche C, Ren J, Kim M, Walker L, Maurer BT, Knolhoff DR, Shick KM. Evaluation of Intraoperative, Local Site Injections of Liposomal Bupivacaine as an Alternative to Standard Local Anesthetics in Patients Undergoing Total Hip Arthroplasty. Hosp Pharm. 2020;55(4):273-8.

194. Ramkumar PN, Haeberle HS, Ramanathan D, Cantrell WA, Navarro SM, Mont MA, Bloomfield M, Patterson BM. Remote Patient Monitoring Using Mobile Health for Total Knee Arthroplasty: Validation of a Wearable and Machine Learning-Based Surveillance Platform. J Arthroplasty. 2019;34(10):2253-9.

195. Remérand F, Le Tendre C, Baud A, Couvret C, Pourrat X, Favard L, Laffon M, Fusciardi J. The early and delayed analgesic effects of ketamine after total hip arthroplasty: a prospective, randomized, controlled, double-blind study. Anesth Analg. 2009;109(6):1963-71.

196. Reuben SS, Buvenandran A, Katz B, Kroin JS. A prospective randomized trial on the role of perioperative celecoxib administration for total knee arthroplasty: improving clinical outcomes. Anesth Analg. 2008;106(4):1258-64, table of contents.

197. Reuben SS, Ekman EF, Charron D. Evaluating the analgesic efficacy of administering celecoxib as a component of multimodal analgesia for outpatient anterior cruciate ligament reconstruction surgery. Anesth Analg. 2007;105(1):222-7.

198. Rodríguez-Vela J, Lobo-Escolar A, Joven-Aliaga E, Herrera A, Vicente J, Suñén E, Loste A, Tabuenca A. Perioperative and short-term advantages of mini-open approach for lumbar spinal fusion. Eur Spine J. 2009;18(8):1194-201.

199. Roy CFA, A. J.; Davison, P. A Review of Wound Infusion With Local Anesthetics in Plastic Surgery. Annals of plastic surgery. 2019.

200. Salinas FV, Liu SS, Mulroy MF. The effect of single-injection femoral nerve block versus continuous femoral nerve block after total knee arthroplasty on hospital length of stay and long-term functional recovery within an established clinical pathway. Anesth Analg. 2006;102(4):1234-9.

201. Smith EB, Kazarian GS, Maltenfort MG, Lonner JH, Sharkey PF, Good RP. Periarticular Liposomal Bupivacaine Injection Versus Intra-Articular Bupivacaine Infusion Catheter for Analgesia After Total Knee Arthroplasty: A Double-Blinded, Randomized Controlled Trial. J Bone Joint Surg Am. 2017;99(16):1337-44.

202. Stanley B, Jackson A, Norman A, Collins L, Bonomo A, Bonomo Y. Opioid prescribing improvement in orthopaedic specialty unit in a tertiary hospital: a retrospective audit of hospital discharge data pre- and post-intervention for better opioid prescribing practice. ANZ J Surg. 2019;89(10):1302-7.

203. Stepan JG, Lovecchio FC, Premkumar A, Kahlenberg CA, Albert TJ, Baurley JW, Nwachukwu BU. Development of an Institutional Opioid Prescriber Education Program and Opioid-Prescribing Guidelines: Impact on Prescribing Practices. J Bone Joint Surg Am. 2019;101(1):5-13.

204. Szeverenyi C, Kekecs Z, Johnson A, Elkins G, Csernatony Z, Varga K. The Use of Adjunct Psychosocial Interventions Can Decrease Postoperative Pain and Improve the Quality of Clinical Care in Orthopedic Surgery: A Systematic Review and Meta-Analysis of Randomized Controlled Trials. J Pain. 2018;19(11):1231-52.

205. Tashjian RZ, Banerjee R, Bradley MP, Alford W, Fadale PD. Zolpidem reduces postoperative pain, fatigue, and narcotic consumption following knee arthroscopy: a prospective randomized placebo-controlled double-blinded study. J Knee Surg. 2006;19(2):105-11.

206. Tetsunaga T, Tetsunaga T, Fujiwara K, Endo H, Ozaki T. Combination Therapy with Continuous Three-in-One Femoral Nerve Block and Periarticular Multimodal Drug Infiltration after Total Hip Arthroplasty. Pain Res Manag. 2016;2016:1425201.

207. Wang L, Bauer M, Curry R, Larsson A, Sessler DI, Eisenach JC. Intrathecal ketorolac does not improve acute or chronic pain after hip arthroplasty: a randomized controlled trial. J Anesth. 2014;28(5):790-3.

208. Wong EM, Chan SW, Chair SY. Effectiveness of an educational intervention on levels of pain, anxiety and self-efficacy for patients with musculoskeletal trauma. J Adv Nurs. 2010;66(5):1120-31.

209. Wrotslavsky P. Pain Reduction with Negative Pressure on Surgical Site Incisions. Surg Technol Int. 2018;33:47-51.

210. Wu N, Yan S, Wang X, Lv C, Wang J, Zheng Q, Feng Y, Yang Y. A prospective, single-blind randomised study on the effect of intercostal nerve protection on early post-thoracotomy pain relief. Eur J Cardiothorac Surg. 2010;37(4):840-5.

211. Zhang SP, J.; Nantha-Aree, M.; Buckley, N.; Shahzad, U.; Cheng, J.; Debeer, J.; Winemaker, M.; Wismer, D.; Punthakee, D.; et al.,. Reanalysis of morphine consumption from two randomized controlled trials of gabapentin using longitudinal statistical methods. Journal of pain research. 2015;8.

212. Zhou Y, Yang TB, Wei J, Zeng C, Li H, Yang T, Lei GH. Single-dose intra-articular ropivacaine after arthroscopic knee surgery decreases post-operative pain without increasing side effects: a systematic review and meta-analysis. Knee Surg Sports Traumatol Arthrosc. 2016;24(5):1651-9.

213. Van Horne A, Van Horne J. Presurgical optimization and opioid-minimizing enhanced recovery pathway for ambulatory knee and hip arthroplasty: postsurgical opioid use and clinical outcomes. Arthroplast Today. 2020;6(1):71-6.

214. Leas DP, Connor PM, Schiffern SC, D'Alessandro DF, Roberts KM, Hamid N. Opioid-free shoulder arthroplasty: a prospective study of a novel clinical care pathway. J Shoulder Elbow Surg. 2019;28(9):1716-22.

215. King CA, Landy DC, Bradley AT, Scott B, Curran J, Devanagondi S, Balach T, Mica MC. Opioid Naive Surgeons and Opioid-Tolerant Patients: Can Education Alter Prescribing Patterns to Total Knee Arthroplasty Patients? J Knee Surg. 2021;34(10):1042-7.

216. Aran F, Wang KY, Rosas S, Danelson KA, Emory CL. The Effect of the Strengthen Opioid Misuse Prevention Act on Opiate Prescription Practices Within the Orthopaedic Surgery Department of an Academic Medical Center. J Am Acad Orthop Surg Glob Res Rev. 2020;4(3).

217. Anthony CA, Rojas EO, Keffala V, Glass NA, Shah AS, Miller BJ, Hogue M, Willey MC, Karam M, Marsh JL. Acceptance and Commitment Therapy Delivered via a Mobile Phone Messaging Robot to Decrease Postoperative Opioid Use in Patients With Orthopedic Trauma: Randomized Controlled Trial. J Med Internet Res. 2020;22(7):e17750.

218. Hartwell MJ, Selley RS, Terry MA, Tjong VK. Can We Eliminate Opioid Medications for Postoperative Pain Control? A Prospective, Surgeon-Blinded, Randomized Controlled Trial in Knee Arthroscopic Surgery. Am J Sports Med. 2020;48(11):2711-7.

219. Pannu TS, Villa JM, Fleites J, Patel PD, Higuera CA, Riesgo AM. Florida State Opioid Prescription Restriction Law: Impact on Opioid Utilization After Total Joint Arthroplasty. J Arthroplasty. 2021;36(8):2742-5.

220. Rucinski K, Cook JL. Effects of preoperative opioid education on postoperative opioid use and pain management in orthopaedics: A systematic review. J Orthop. 2020;20:154-9.

221. Chen EY, Betancourt L, Li L, Trucks E, Marcantonio A, Tornetta P, 3rd. Standardized, Patient-specific, Postoperative Opioid Prescribing After Inpatient Orthopaedic Surgery. J Am Acad Orthop Surg. 2020;28(7):e304-e18.

222. Hannon CP, Fillingham YA, Nam D, Courtney PM, Curtin BM, Vigdorchik JM, Buvanendran A, Hamilton WG, Della Valle CJ. Opioids in Total Joint Arthroplasty: The Clinical Practice Guidelines of the American Association of Hip and Knee Surgeons, American Society of Regional Anesthesia and Pain Medicine, American Academy of Orthopaedic Surgeons, Hip Society, and Knee Society. J Arthroplasty. 2020;35(10):2709-14.

Wrong patient population

223. Aghayev E, Etter C, Bärlocher C, Sgier F, Otten P, Heini P, Hausmann O, Maestretti G, Baur M, Porchet F, et al. Five-year results of lumbar disc prostheses in the SWISSspine registry. Eur Spine J. 2014;23(10):2114-26.

224. Aghayev E, Bärlocher C, Sgier F, Hasdemir M, Steinsiepe KF, Wernli F, Porchet F, Hausmann O, Ramadan A, Maestretti G, et al. Five-year results of cervical disc prostheses in the SWISSspine registry. Eur Spine J. 2013;22(8):1723-30.

225. Aghayev EM, P.; Röder, C. Intermediate clinical results of cervical disc prostheses in the SWISSspine registry. European Spine Journal. 2010;19(11).

226. Atchison JWB, A.; Gagnon, C. M.; Margolis, S.; Calisoff, R. L. Reduction in opioid use for injured workers following completion of a 4-week interdisciplinary pain/functional restoration program. PM and R. 2017;9(9).

227. Beaussier M, Parc Y, Guechot J, Cachanado M, Rousseau A, Lescot T. Ropivacaine preperitoneal wound infusion for pain relief and prevention of incisional hyperalgesia after laparoscopic colorectal surgery: a randomized, triple-arm, double-blind controlled evaluation vs intravenous lidocaine infusion, the CATCH study. Colorectal Dis. 2018;20(6):509-19.

228. Beyerlein JH-A, N.; Noriega, D.; Ardura, F.; Hassel, F.; Barreau, X. Clinical outcome after the use of a new cranio-caudal expandable implant for vertebral compression fracture treatment. 1-year results from a prospective multicenter study. European Spine Journal 2012;21(11).

229. Blasco J, Martinez-Ferrer A, Macho J, San Roman L, Pomés J, Carrasco J, Monegal A, Guañabens N, Peris P. Effect of vertebroplasty on pain relief, quality of life, and the incidence of new vertebral fractures: a 12-month randomized follow-up, controlled trial. J Bone Miner Res. 2012;27(5):1159-66.

230. Block CC, L.; Andrews, S.; McConley, R.; Doleys, D. Improvements in objective and subjective functioning following completion of an interdisciplinary pain rehabilitation program. Journal of Pain. 2011;12(4).

231. Bollag L, Richebe P, Siaulys M, Ortner CM, Gofeld M, Landau R. Effect of transversus abdominis plane block with and without clonidine on post-cesarean delivery wound hyperalgesia and pain. Reg Anesth Pain Med. 2012;37(5):508-14.

232. Brose SW, Schneck H, Bourbeau DJ. An Interdisciplinary Approach to Reducing Opioid Prescriptions to Patients with Chronic Pain in a Spinal Cord Injury Center. Pm r. 2019;11(2):135-41.

233. Cohen SP, Galvagno SM, Plunkett A, Harris D, Kurihara C, Turabi A, Rehrig S, Buckenmaier CC, 3rd, Chelly JE. A multicenter, randomized, controlled study evaluating preventive etanercept on postoperative pain after inguinal hernia repair. Anesth Analg. 2013;116(2):455-62.

234. Dalle Ore CL, Ames CP, Deviren V, Lau D. Outcomes Following Single-Stage Posterior Vertebral Column Resection for Severe Thoracic Kyphosis. World Neurosurg. 2018;119:e551-e9.

235. Derry S, Wiffen PJ, Kalso EA, Bell RF, Aldington D, Phillips T, Gaskell H, Moore RA. Topical analgesics for acute and chronic pain in adults - an overview of Cochrane Reviews. Cochrane Database Syst Rev. 2017;5(5):Cd008609.

236. Lavand'homme P, De Kock M, Waterloos H. Intraoperative epidural analgesia combined with ketamine provides effective preventive analgesia in patients undergoing major digestive surgery. Anesthesiology. 2005;103(4):813-20.

237. Mpotsaris A, Abdolvahabi R, Hoffleith B, Nickel J, Harati A, Loehr C, Gerdes CH, Hennigs S, Weber W. Percutaneous vertebroplasty in vertebral compression fractures of benign or malignant origin: a prospective study of 1188 patients with follow-up of 12 months. Dtsch Arztebl Int. 2011;108(19):331-8.

238. Pedicelli A, Rollo M, Piano M, Grattacaso G, Colosimo C, Bonomo L. Percutaneous vertebroplasty: optimizing the procedure after treatment of 250 vertebral levels under fluoroscopic guidance. Radiol Med. 2009;114(7):1141-58.

239. Pesonen A, Suojaranta-Ylinen R, Hammarén E, Kontinen VK, Raivio P, Tarkkila P, Rosenberg PH. Pregabalin has an opioid-sparing effect in elderly patients after cardiac surgery: a randomized placebo-controlled trial. Br J Anaesth. 2011;106(6):873-81.

240. Zolin SJ, Ho VP, Young BT, Harvey AR, Beel KT, Tseng ES, Brown LR, Claridge JA. Opioid prescribing in minimally injured trauma patients: Effect of a state prescribing limit. Surgery. 2019;166(4):593-600.

241. Kwok AK, O'Hara NN, Pollak AN, O'Hara LM, Herman A, Welsh CJ, Slobogean GP. Are injured workers with higher rehabilitation service utilization less likely to be persistent opioid users? A cross-sectional study. BMC Health Serv Res. 2019;19(1):32.

242. Qaseem A, McLean RM, O'Gurek D, Batur P, Lin K, Kansagara DL, Cooney TG, Forciea MA, Crandall CJ, Fitterman N, et al. Nonpharmacologic and Pharmacologic Management of Acute Pain From Non-Low Back, Musculoskeletal Injuries in Adults: A Clinical Guideline From the American College of Physicians and American Academy of Family Physicians. Ann Intern Med. 2020;173(9):739-48.

Wrong study design

243. Rhon DI, Snodgrass SJ, Cleland JA, Greenlee TA, Sissel CD, Cook CE. Comparison of Downstream Health Care Utilization, Costs, and Long-Term Opioid Use: Physical Therapist Management Versus Opioid Therapy Management After Arthroscopic Hip Surgery. Phys Ther. 2018;98(5):348-56.

244. Alencar de Castro RJ, Leal PC, Sakata RK. Pain management in burn patients. Braz J Anesthesiol. 2013;63(1):149-53.

245. Anastase DM, Florescu SC, Munteanu AM, Stoica I, Antonescu D. The influence of the analgesic model on postoperative pain in major knee surgery. Chirurgia (Bucur). 2013;108(6):764-9.

246. Andersen L, Kehlet H. Analgesic efficacy of local infiltration analgesia in hip and knee arthroplasty: a systematic review. Br J Anaesth. 2014;113(3):360-74.

247. Bhashyam AR, Keyser C, Miller CP, Jacobs J, Bluman E, Smith JT, Chiodo C. Prospective Evaluation of Opioid Use After Adoption of a Prescribing Guideline for Outpatient Foot and Ankle Surgery. Foot Ankle Int. 2019;40(11):1260-6.

248. Bowlby MA, Crawford ME. Opioid Crisis and Acute Pain Management After Foot and Ankle Surgery. Clin Podiatr Med Surg. 2019;36(4):695-705.

249. Carlos Rodriguez-Merchan E, Vaquero-Picado A, Ruiz-Perez JS. Opioid-Free Total Knee Arthroplasty? Local Infiltration Analgesia Plus Multimodal Blood-Loss Prevention Make it Possible. Hss j. 2019;15(1):17-9.

250. Cibrian K. Nondrug Interventions Reduce Pain and Opioid Use After Total Knee Arthroplasty. Am J Nurs. 2017;117(11):62.

251. Coghlan JA, Forbes A, Bell SN, Buchbinder R. Efficacy and safety of a subacromial continuous ropivacaine infusion for post-operative pain management following arthroscopic rotator cuff surgery: a protocol for a randomised double-blind placebo-controlled trial. BMC Musculoskelet Disord. 2008;9:56.

252. Crisologo PA, Monson EK, Atway SA. Gabapentin as an Adjunct to Standard Postoperative Pain Management Protocol in Lower Extremity Surgery. J Foot Ankle Surg. 2018;57(4):781-4.

253. Degenhardt L, Grebely J, Stone J, Hickman M, Vickerman P, Marshall BDL, Bruneau J, Altice FL, Henderson G, Rahimi-Movaghar A, et al. Global patterns of opioid use and dependence: harms to populations, interventions, and future action. Lancet. 2019;394(10208):1560-79.

254. Faour MA, J. T.; Haas, A. R.; Woods, S. T.; Ahn, U. M.; Ahn, N. U. Preoperative duration of opioid use and return to work (RTW) rates after single-level cervical fusion in workers' compensation (WC) setting. Spine Journal. 2015;15(10).

255. Fuentes S, Metellus P, Fondop J, Pech-Gourg G, Dufour H, Grisoli F. [Percutaneous pedicle screw fixation and kyphoplasty for management of thoracolumbar burst fractures]. Neurochirurgie. 2007;53(4):272-6.

256. Fuzier R, Rousset J, Bataille B, Salces-y-Nédéo A, Maguès JP. One half of patients reports persistent pain three months after orthopaedic surgery. Anaesth Crit Care Pain Med. 2015;34(3):159-64.

257. Gabriel RA, Swisher MW, Sztain JF, Furnish TJ, Ilfeld BM, Said ET. State of the art opioid-sparing strategies for post-operative pain in adult surgical patients. Expert Opin Pharmacother. 2019;20(8):949-61.

258. Gatewood CT, Tran AA, Dragoo JL. The efficacy of post-operative devices following knee arthroscopic surgery: a systematic review. Knee Surg Sports Traumatol Arthrosc. 2017;25(2):501-16.

259. Golladay GJ, Balch KR, Dalury DF, Satpathy J, Jiranek WA. Oral Multimodal Analgesia for Total Joint Arthroplasty. J Arthroplasty. 2017;32(9s):S69-s73.

260. Gore M, Brix Finnerup N, Sadosky A, Tai KS, Cappelleri JC, Mardekian J, George Rice C, Nieshoff E. Pain-related pharmacotherapy, healthcare resource use and costs in spinal cord injury patients prescribed pregabalin. Spinal Cord. 2013;51(2):126-33.

261. Hanna MN, Speed TJ, Shechter R, Grant MC, Sheinberg R, Goldberg E, Campbell CM, Theodore N, Koch CG, Williams K. An Innovative Perioperative Pain Program for Chronic Opioid Users: An Academic Medical Center's Response to the Opioid Crisis. Am J Med Qual. 2019;34(1):5-13.

262. Hansen CA, Inacio MCS, Pratt NL, Roughead EE, Graves SE. Chronic Use of Opioids Before and After Total Knee Arthroplasty: A Retrospective Cohort Study. J Arthroplasty. 2017;32(3):811-7.e1.

263. Howard ML, Isaacs AN, Nisly SA. Continuous Infusion Nonsteroidal Anti-Inflammatory Drugs for Perioperative Pain Management. J Pharm Pract. 2018;31(1):66-81.

264. Karamchandani K, Klick JC, Linskey Dougherty M, Bonavia A, Allen SR, Carr ZJ. Pain management in trauma patients affected by the opioid epidemic: A narrative review. The journal of trauma and acute care surgery. 2019;87(2):430-9.

265. Katz J, Weinrib A, Fashler SR, Katznelzon R, Shah BR, Ladak SS, Jiang J, Li Q, McMillan K, Santa Mina D, et al. The Toronto General Hospital Transitional Pain Service: development and implementation of a multidisciplinary program to prevent chronic postsurgical pain. J Pain Res. 2015;8:695-702.

266. Kendall MC, Castro Alves LJ. Pain after ortho-plastic reconstruction of lower limb injuries: The importance of standardizing analgesic management. Injury. 2018;49(6):1239-40.

267. Kluger MT, Lewis G, Rice D, McNair P. Psychological rather than pharmacological interventions for effective prevetion of pain after knee joint replacement? Br J Anaesth. 2016;116(1):150.

268. Kopp SL, Lanier WL. Pain Management in Patients Undergoing Radical Pelvic Exenteration Surgery: Opioid Stewardship and the Development of Evidence-Based Alternatives. Dis Colon Rectum. 2018;61(3):267-70.

269. Kumar K, Kirksey MA, Duong S, Wu CL. A Review of Opioid-Sparing Modalities in Perioperative Pain Management: Methods to Decrease Opioid Use Postoperatively. Anesth Analg. 2017;125(5):1749-60.

270. Labrum JTt, Ilyas AM. The Opioid Epidemic: Postoperative Pain Management Strategies in Orthopaedics. JBJS Rev. 2017;5(8):e14.

271. Leng Z, He X. A supplementary study from China: the use of pain medications after operative treatment of an ankle fracture. Injury. 2013;44(11):1656-7.

272. Lespasio MJ, Guarino AJ, Sodhi N, Mont MA. Pain Management Associated with Total Joint Arthroplasty: A Primer. Perm J. 2019;23.

273. Lindestrand AG, Christiansen ML, Jantzen C, van der Mark S, Andersen SE. Opioids in hip fracture patients: an analysis of mortality and post hospital opioid use. Injury. 2015;46(7):1341-5.

274. Liu XL, Tan JY, Molassiotis A, Suen LK, Shi Y. Acupuncture-Point Stimulation for Postoperative Pain Control: A Systematic Review and Meta-Analysis of Randomized Controlled Trials. Evid Based Complement Alternat Med. 2015;2015:657809.

275. Lovecchio F, Derman P, Stepan J, Iyer S, Christ A, Grimaldi P, Kumar K, Ranawat A, Taylor SA. Support for Safer Opioid Prescribing Practices: A Catalog of Published Use After Orthopaedic Surgery. J Bone Joint Surg Am. 2017;99(22):1945-55.

276. McAnally H. Rationale for and approach to preoperative opioid weaning: a preoperative optimization protocol. Perioper Med (Lond). 2017;6:19.

277. Morris BJ, Zumsteg JW, Archer KR, Cash B, Mir HR. Narcotic Use and Postoperative Doctor Shopping in the Orthopaedic Trauma Population. J Bone Joint Surg Am. 2014;96(15):1257-62.

278. Oseka L, Pecka S. Anesthetic Management in Early Recovery After Surgery Protocols for Total Knee and Total Hip Arthroplasty. Aana j. 2018;86(1):32-9.

279. Oyler DR, Parli SE, Bernard AC, Chang PK, Procter LD, Harned ME. Nonopioid management of acute pain associated with trauma: Focus on pharmacologic options. J Trauma Acute Care Surg. 2015;79(3):475-83.

280. Parker Vail T. Preoperative pain management decisions impact outcome after total knee arthroplasty-implications for opiate use: commentary on an article by Michael G. Zywiel, MD, et al.: "Chronic opioid use prior to total knee arthroplasty". J Bone Joint Surg Am. 2011;93(21):e1291-1.

281. Parvizi J. Pain management following total joint arthroplasty: making strides. J Bone Joint Surg Am. 2012;94(16):1441.

282. Reinke CE, Dodgion C. Opioids After Orthopedic Injuries: Just the Beginning. J Surg Res. 2019;240:241.

283. Rose P, Ramlogan R, Sullivan T, Lui A. Serratus anterior plane blocks provide opioid-sparing analgesia in patients with isolated posterior rib fractures: a case series. Can J Anaesth. 2019;66(10):1263-4.

284. Saigal AN, Jones HM. Interdisciplinary Mitigation of Opioid Misuse in Musculoskeletal Patients. Hss j. 2019;15(1):72-5.

285. Saloner B, McGinty EE, Beletsky L, Bluthenthal R, Beyrer C, Botticelli M, Sherman SG. A Public Health Strategy for the Opioid Crisis. Public Health Rep. 2018;133(1_suppl):24s-34s.

286. Secrist ES, Freedman KB, Ciccotti MG, Mazur DW, Hammoud S. Pain Management After Outpatient Anterior Cruciate Ligament Reconstruction: A Systematic Review of Randomized Controlled Trials. Am J Sports Med. 2016;44(9):2435-47.

287. Seymour RB, Ring D, Higgins T, Hsu JR. Leading the Way to Solutions to the Opioid Epidemic: AOA Critical Issues. J Bone Joint Surg Am. 2017;99(21):e113.

288. Sing DC, Barry JJ, Cheah JW, Vail TP, Hansen EN. Long-Acting Opioid Use Independently Predicts Perioperative Complication in Total Joint Arthroplasty. J Arthroplasty. 2016;31(9 Suppl):170-4.e1.

289. Singh PM, Borle A, Trikha A, Michos L, Sinha A, Goudra B. Role of Periarticular Liposomal Bupivacaine Infiltration in Patients Undergoing Total Knee Arthroplasty-A Meta-analysis of Comparative Trials. J Arthroplasty. 2017;32(2):675-88.e1.

290. Soffin EM, Wu CL. Regional and Multimodal Analgesia to Reduce Opioid Use After Total Joint Arthroplasty: A Narrative Review. Hss j. 2019;15(1):57-65.

291. Steadman JL, Jones J. Nerve Blockade and Chronic Opiate Use After Orthopedic Surgery. Anesth Analg. 2018;126(2):731-2.

292. Stewart TM, Pulos N, Curry TB, Gazelka HM, Brown MJ, Shin AY. Team Approach: Multimodal Perioperative Pain Management in Upper-Extremity Surgery: Combating the Opioid Epidemic. JBJS Rev. 2018;6(8):e5.

293. Tedesco D, Gori D, Desai KR, Asch S, Carroll IR, Curtin C, McDonald KM, Fantini MP, Hernandez-Boussard T. Drug-Free Interventions to Reduce Pain or Opioid Consumption After Total Knee Arthroplasty: A Systematic Review and Meta-analysis. JAMA Surg. 2017;152(10):e172872.

294. von Dincklage F, Jakuscheit A, Weth J, Lichtner G, Jurth C, Rehberg-Klug B. Higher doses of intraoperative analgesia are associated with lower levels of persistent pain and less analgesic consumption six months after total hip arthroplasty. Eur J Pain. 2018;22(4):691-9.

295. von Dincklage FJ, A.; Weth, J.; Lichtner, G.; Jurth, C.; Rehberg-Klug, B. Comment on a paper by Von Dincklage et al. entitled 'Higher doses of intraoperative analgesia are associated with lower levels of persistent pain and less analgesic consumption six months after total hip arthroplasty' Reply. European Journal of Pain 2018;22(4).

296. Wang C, Zhang J. Reply letter to: "Commentary on: Ketamine reduces pain and opioid consumption after total knee arthroplasty: A meta-analysis of randomized controlled studies". Int J Surg. 2019;72:43-4.

297. Wang J, Liu GT, Mayo HG, Joshi GP. Pain Management for Elective Foot and Ankle Surgery: A Systematic Review of Randomized Controlled Trials. J Foot Ankle Surg. 2015;54(4):625-35.

298. Warrender WJ, Syed UAM, Hammoud S, Emper W, Ciccotti MG, Abboud JA, Freedman KB. Pain Management After Outpatient Shoulder Arthroscopy: A Systematic Review of Randomized Controlled Trials. Am J Sports Med. 2017;45(7):1676-86.

299. Xue FS, Liu GP, Sun C, Yang GZ. Comparing Local Infiltration and Continuous Femoral Nerve Block for Pain Relief After Total Knee Arthroplasty. J Arthroplasty. 2016;31(5):1133-4.

300. Clarke H, Azargive S, Montbriand J, Nicholls J, Sutherland A, Valeeva L, Boulis S, McMillan K, Ladak SSJ, Ladha K, et al. Opioid weaning and pain management in postsurgical patients at the Toronto General Hospital Transitional Pain Service. Canadian Journal of Pain. 2018;2(1):236-47.

301. Abid Azam M, Weinrib AZ, Montbriand J, Burns LC, McMillan K, Clarke H, Katz J. Acceptance and Commitment Therapy to manage pain and opioid use after major surgery: Preliminary outcomes from the Toronto General Hospital Transitional Pain Service. Canadian Journal of Pain. 2017;1(1):37-49.

302. Agarwal AK, Lee D, Ali Z, Sennett B, Xiong R, Hemmons J, Spencer E, Abdel-Rahman D, Kleinman R, Lacko H, et al. Patient-Reported Opioid Consumption and Pain Intensity After Common Orthopedic and Urologic Surgical Procedures With Use of an Automated Text Messaging System. JAMA Netw Open. 2021;4(3):e213243.

303. Buys MJ, Bayless K, Romesser J, Anderson Z, Patel S, Zhang C, Presson AP, Beckstrom J, Brooke BS. Multidisciplinary Transitional Pain Service for the Veteran Population. Fed Pract. 2020;37(10):472-8.

304. Randall DJ, Vanood A, Jee Y, Moore DD. National and State Level Opioid-Restricting Legislation in Total Joint Arthroplasty: A Systematic Review. J Arthroplasty. 2021.

305. Brown-Taylor L, Beckner A, Scaff KE, Fritz JM, Buys MJ, Patel S, Bayless K, Brooke BS. Relationships between physical therapy intervention and opioid use: A scoping review. PM R. 2021.
