# Supplementary material for: Strategies aimed at preventing long-term opioid use in trauma and orthopaedic surgery: a scoping review
Source: BMC Musculoskelet Disord. 2022 Mar 11;23:238. doi: 10.1186/s12891-022-05044-y (PMC8917706; doi:10.1186/s12891-022-05044-y)
Supplement: Supplementary file 4 — Additional file 4. Risk factor for chronic opioid use in included studies by type of strategies. [file 12891_2022_5044_MOESM4_ESM.docx]

Supplemental Digital file 4. Risk factor for chronic opioid use in included studies by type of strategies

| **First author, year,** | **Risk factor for chronic opioid use** | | | | |
| --- | --- | --- | --- | --- | --- |
| **Studies including trauma patients** | | | | | |
| **System-based** | | | | | |
| Chambers 2021^(96)^ | Chronic daily users of opioids were excluded | | |  | |
| Chen 2020^(77)^ | NS | | |  | |
| Reid 2020^(87)^ | *Opioid-tolerant*: I: 5.0%; C: 4.4%  *Recent benzodiazepine use*: I: 7.9%; C: 6.4% | | |  | |
| Wyles 2020^(97)^ | *Preoperative opioid use*:  Naïve I: 72.0%; C: 72.0%  Tolerant: I: 28.0%; C: 28.0% | | | *Anxiety diagnosis:* I: 10.0%; C: 10.0%  *Depression diagnosis:* I: 16.0%; C: 15% | |
| Choo 2019^(79)^ | Depression: I: 4.7%; C: 5.2% | | | (Patients taking opioids before surgery were excluded) | |
| Reid 2019^(86)^ | *Opioid tolerant*: I: 13.7%; C:13.6%  *Recent anxiolytic use*: I: 9.0%; C: 8.0% | | | | |
| Young 2019^(93)^ | *Alcohol abuse*: I: 14.0%; C :10.6% | | | | |
| Earp 2018^(80)^ | Patients on chronic opioid therapy were excluded. | | | | |
| **Pharmacological** | | | | | |
| Cunningham 2021^(98)^ | *Smoking: I:*11.8%; C: 27.3%*  *Preo-perative opioid usage*: I: 14.1%; C: 10.8% | |  | | |
| Cunningham 2021^(99)^ | *Smoking: I:*21.5%; C: 26.5%  *Preo-perative opioid usage*: I: 9.0%; C: 8.0% | |  | | |
| Bhashyam 2018^(63)^ | *Prior opioid use*: I1: 12.9%; I2: 13.0%; C: 7.0%*  *PROMIS anxiety score*^1^: I1: 6.8; I2: 8.4; C: 6.1*  *Pain catastrophizing score*^2^: I1: 18.9; I2: 21.3; C: 15.8* | | *Benzodiazepine use*: I1: 12.5%; I2: 10.1%; C: 7.5%  *Antidepressant use*: I1: 23.7%; I2:26.1%; C: 16.1% | | |
| Radi 2017^(94)^ | *Tobacco use***:** I: 36.5%; C: 40.9%  Patients with an history of opioid dependence, current chronic opioid use (daily use > 4 weeks) were excluded. | | | | |
| Yazdani 2016^(75)^ | NS (patients with preoperative opioid use or dependency, psychological disease, mental disorder with loss of normal personality and reality, mental disturbance, suicidal thoughts, and alcoholism were excluded). | | | | |
| Gray 2011^(66)^ | Patients with significant history of psychiatric illness or substance abuse were excluded. | | | | |
| **Educational** | | | | | |
| Bérubé 2021^(100)^ | *Annual income < 50 000* : I: 60.0%; C: 60%  *History of substance abuse* :  *Opioids*: I : 9.0%; C : 16.0%  *Benzodiazepine :* I : 9.0%; C : 20.0%  *Cannabis :* I : 55.0%; C : 33.0%  *Cocaine*: I : 19.0%; C : 5.0%  *Tobacco :* I : 56.0%; C : 43.0%  *Alcohol:* I: 20.0%; C: 19.0% | | *Pain catastrophizing score > 20*: I:36.0%; C: 21.0%  *Anxiety disorder Score > 10*: I: 24.0%; C: 17%  *Depression disorder Score > 10*: I: 12.0%; C: 4.0%  *Pain self-efficacy Score < 17*: I: 16.0%; C: 4%  All patients have at least one risk factor for chronic opioid use.  Patients with severe psychiatric disorders were excluded. | | |
| Syed 2018^(53)^ | *Tobacco use*: I: 8.8%; C: 21.2%  *Anxiety/depression*: I: 30.9%; C:25.8% | | *Opioid risk* (high to moderate): I: 15%; C:12%  *Curent opioid use*: I: 32.3%; C: 50% | | |
| Stanek 2015^(52)^ | NS | | | | |
| Holman 2014^(5)^ | Previous opiate use (> 1 times): I: 11.0%; C:13.0% | | | | |
| **Multimodal** | | | | | |
| Singer 2021^(101)^ | NS | | | | |
| **Alternative** | | | | | |
| Crawford 2019^(55)^ | Patients with chronic (>30 days) daily opiate use were excluded. | | | | |
| **Psychological** | | | | | |
| **Studies including non-trauma surgical patients: orthopeadic, spine and general** | | | | | |
| **System-based** | | | | | |
| Chalmers 2021^(102)^ | *Chronic pain syndrome*: total: 11.5%  *Preoperative tramadol use*: total: 12.8%  *Preoperative benzodiazepine use:* total: 10.8 % | *Anxiety or depression*: total: 24.9%  *Current smoker*: total: 5.7 % | | |  |
| Cunningham 2021^(103)^ | *Current smoking:* I1: 8.8%; I2: 7.2%; C: 8.6 %  *Preoperative opioid prescription:* I1: 22.0%; I2: 21%; C:27%* |  | | |  |
| Raji 2021^(104)^ | *Tobacco use* : I: 39.6%; C: 55.6%*  Alcohol use: I: 59.1%; C: 51.5%  Chronic pain: I: 10.2%; C: 14.1% | *Prescriptions filled within 30 days prior to surgery:*  Opioid : total: I: 21,7%: C: 29.3%  Benzodiazepine: total: I: 7.7%; C: 12.1% | | |  |
| Sabesan 2021^(105)^ | *Smoking status*: *  Never: I: 58.9%; C: 66.7%  Current: I: 5.4%; C: 3.4%  Former: I: 35.7%; C: 29.9% |  | | |  |
| Eley 2020^(81)^ | Chronic opioid users were excluded. | | | | |
| Joo 2020^(83)^ | *Psychiatric comorbidity*: I: 60.5%; C: 53.3% *Prescribed preoperative opioids*: I: 23.7%; C: 35.6%  *Substance abuse disorder*: I: 39.5%; C: 46.7% | | | | |
| Tamboli 2020^(89)^ | *Psychiatric comorbidity*: I: 33.0%; C: 40.0% *Preoperative opioid prescribed*: I: 33.0%; C: 44.0%  *Substance abuse disorder*: I: 12.0%; C: 8.0% | | | | |
| Whale 2020^(91)^ | NS | | | | |
| Chen 2019^(78)^ | *Alcohol abuse*: I: 23.6%; C: 21.3%*  *Drug abuse*: I: 18.5%; C: 17.4%*  *Psychoses*: I: 9.5%; C: 9.6%  *Depression*: I: 53.8%; C: 51.4%* | | *Preoperative opioid use*:  Chronic: I: 25.5%; C: 32.2%*  Intermittent: I: 46.2%; C: 38.7%* | | |
| Holte 2019^(82)^ | *Prior opiate use*: I: 37.6%; C: 35.1%  *Prolonged opiate use*: I:25.6%; C: 29.4%  *PROMIS Mental Health*^1^: I: 48.6 (8.4); C: 50.1 (9.1) | | Patients previously requiring pain management specialists for management of chronic pain or long-term opioid prescriptions were excluded. | | |
| Padilla 2019^(84)^ | *Smoking status*:  Current: I: 4.3%; C: 4.3%  Former: I: 40.6%; C: 42.5% | | | | |
| Reid 2019^(88)^ | *Opioid tolerant*: I: 16.7%; C:18.4%  *Recent benzodiazepine use*: I: 10.9%; C: 7.2%* | | | | |
| Reid 2019^(85)^ | *Preoperative opioid use*:  30 days: I: 30.0%; C: 37.6%  90 days: I: 42.7%; C: 50.5% | | *Preoperative benzodiazepine use*:  30 days: I: 14.6%; C: 18.8%  90 days: I: 20.0%; C: 26.7% | | |
| Vaz 2019^(90)^ | Patients with history of opiate dependency or narcotic abuse were excluded. | | | | |
| Wyles 2019^(92)^ | *Psychiatric illness*: I: 16.8%; C: 16.0%  *Outpatient benzodiazepines*: I: 3.7%; C: 3.9%  *Current smoker*: I: 6.7%; C: 6.2% | | | | |
| **Pharmacological** | | | | | |
| Burns 2021^(106)^ | *Smoking status*:  Current: I: 21.5%; C: 14.1%  Former: I: 21.5%; C: 24.4% | | *Preoperative narcotic use*: I: 36.7%; C: 33.3%  (Patients with baseline long-acting opioids use were excluded) | | |
| Zhuang 2020^(76)^ | NS (Patients were excluded if addicted to opioids or alcohol or had a history of chronic pain or an unstable psychiatric condition). | | | | |
| Starr 2019^(72)^ | *Depression*: I: 39.7%; C: 39.4%  *PTSD*: I: 21.7%; C: 23.0%  *Tobacco use*: I: 13.6%; C: 16.1%* | | | | |
| Fenten 2018^(65)^ | NS (patients with opioids or anti-neuropathic pain medication use >1 yr, or physical, emotional, or neurological conditions were excluded). | | | | |
| Hah 2018^(67)^ | *Ever use of prescription opioids*: I: 85.5%; C: 82.3%*  *Past 30-d prescription opioid use*: I: 7.7%; C: 8.9%* | | Patients with chronic pain and suicidality risk were excluded. | | |
| Thompson 2018^(74)^ | *Depression*: I: 25.0%; C: 32.1%  *Pre-op narcotic use*: I: 43.8%; C: 57.1%  *Tobacco use*: I: 12.5%; C: 28.6% | | | | |
| Sun 2017^(72)^ | *Alcohol abuse*: I: 0.3%; C: 0.3%  *Depression*: I: 7.2%; C: 6.7%*  *Drug abuse*: I: 0.28%; C: 0.3%  *Antidepressant use*: I: 21.3%; C: 21.0% | | *Benzodiazepines use*: I: 13.7%; C: 13.6%  *Chronic opioid use*: I: 11.0% C: 11.1%  *Intermittent opioid use*: I: 39.8%; C: 39.4% | | |
| Hyer 2015^(69)^ | All patients were free of a syndrome of anxiety or depression and those who presented with psychiatric problems, taking any other psychotropic medication, except for sleep were excluded. | | | | |
| Aguirre 2012^(62)^ | NS | | | | |
| Nader 2012^(70)^ | Patients taking antidepressant or antiepileptic drugs or opioid-tolerant patients were excluded. | | | | |
| Chevet 2011^(64)^ | *Antidepressant use*: I:20.0%; C: 33.0%  *Benzodiazepines use*: I:15.0%; C: 15.0% | | *Morphinomimetics use*: I:42.0%; C: 37.0%    Patients taking > 10 mg/day morphine were excluded. | | |
| Schroer 2011^(71)^ | Patients using opioids preoperatively on a daily basis were excluded. | | | | |
| **Educational** | | | | | |
| Cheesman 2020^(107)^ | *Smoking:* I: I: 8.6%; C: 20.0%  *Anxiety and/or depression:* I: 20.0%; C: 18.6%  *Prior opioid use:* I: 22.9%; C: 30% | | *Risk according to opioid risk tool: Low:* I: 85.7%; C: 88.6; *moderate:*I:5.7%; C: 7.1%; *high:* I: 8.6%; C: 4.3% | | |
| Campbell 2019^(50)^ | *Tobacco use*: I: 7.9%; C: 8.4% | |  | | |
| Smith 2018^(50)^ | *Tobacco use*: I: 4.4%; C: 5.2%*  *Anxiety medication use*: I:7.6%; C: 11.2%  *Antidepressant medication use*: I: 34.6%; C: 42.7% | | *Diagnosis of substance abuse*: I:14.2%; C: 21.3%  *Depression*: I: 27.3%; C: 32.5%  *Anxiety*: I: 16.7%; C: 17.1%* | | |
| **Multimodal** | | | | | |
| Urban 2021^(108)^ | *No prior exposure to opioids : I: 96%; C: 81%* * | |  | | |
| Buys 2020^(109)^ | *History of mental health disorders:* I: 48.2%; C: 47.1%  *History of substance use disorders*: I: 13.4%; C: 14.5% | | *Chronic opioid use :* I: 22.6%; C: 30.8% | | |
| Li 2020^(110)^ | *Current smoker:* I: 5.6%; C: 1.4%  *Alcohol use:* I: 72.5%; C: 57.1% | | Patients with < 3 months preoperative opioid use and/or psychiatric disorder were excluded | | |
| Fleischman 2019^(57)^ | *Pain catastrophizing*^2^:  I1: 11.0 (10.6); I2: 11.1(9.5) C: 11.1 (9.5)*  *Tramadol use*: I1: 8.0%; I2: 9.0%; C: 10.0%*  *Benzodiazepine use*: I1: 4.0%; I2: 6.0%; C: 3.0%* | | Patients with chronic opiate use within 3 months of operation were excluded | | |
| Hannon 2019^(68)^ | *No first-time opioids user*: I: 86.3%; C: 83.9% | |  | | |
| Tan 2018^(58)^ | *Non-smoker*: I: 92.2%; C: 92.2%  *Morphine Equivalent Dose (MED) mg/day* : I: 0.0; C: 0.0; | | | | |
| Dasa 2016^(56)^ | *PROMIS* Anxiety^1^: I: 55.5 (11.1); C: 58.8 (12.9)  *PROMIS Depression*^1^: I: 49.1 (9.1): C: 51.0 (11.2) | | | | |
| **Surgical** | | | | | |
| Bovonratwet 2021^(111)^ | *Smoking status*: *  Nonsmoker:I: 62.9%; C: 48.8%  Current: I: 4.9%; C: 8.5%  Former: I: 32.2%; C: 42.7% | | *Preoperative opioid use*: I: 9.4%; C: 17.07%* | | |
| Varady 2021^(112)^ | *Chronic pain:*  Back: I: 24.1%; C: 24.5%  Neck: I: 8.5%; C: 7.9%  Other: I: 17.2%; C: 18.8%  *Mood disorder*: I: 12.3%; C: 13.3%* | | *Substance use disorder:* I: 2.9%; C: 2.6%  *Suicide or self-harm:* I: 1.5%; C: 1.4%  Patients with < 11 months preoperative opioid use were excluded | | |
| Walega 2019^(61)^ | *Preoperative opioid use*: I: 11.4%; C:18.8% | |  | | |
| Verla 2018^(60)^ | NS | |  | | |
| Della Valle 2010^(59)^ | NS | |  | | |
| **Alternative** | | | | | |
| Collinsworth 2019^(54)^ | NS | |  | | |
| **Psychological** |  | |  | | |
| Hanley 2021^(113)^ | *Tobacco use:* I1: 26.5%; I2: 24.4%; C: 17.9%  *Alcohol use*: I1: 44.1%; I2: 51.1%; C: 38.5%  *Drug use:* I1: 2.9%; I2: - %; C: 2.6%  *Prescription opioid use:* I1: 8.8%; I2: 24.4%; C: 33.3%* | | Patients with a history of substance use disorder are required to  provide a negative drug screen before surgery; patients taking  prescription opioid medication preoperatively are requested to  decrease their dose by at least 50% before surgery | | |
| Hah 2020^(114)^ | *Preoperative opioid use:* I: 24.5% C: 23.6%  *PROMIS* Anxiety^1^: I: 51.0 (6.8); C: 52.1 (8.9)  *PROMIS Depression*^1^: I: 48.2(7.7): C: 47.2 (7.9) | | *Pain catastrophizing*^2^: I: 9.4 (6.5); C: 11.2 (8.0)  Patients receiving car from a pain management doctor or taking around-the-clock prescription opioids in the 30 days preceding surgery and those taking opioids for non-surgical site pain in the 30 days preceding surgery were excluded. | | |
| Dindo 2018^(95)^ | *Other pain conditions*: I: 72.0%; C: 71.0%  *Preoperative opioid use*: I: 30.0%; C: 34.0%  *Opioid risk Tool*^3^: I: 0.5; C: 1,0  *CAGE (substance use disorder)*^4^*:* I: 1; C: 1 | | *BPI severity*^5^: I:4.8; C: 5.0  *BPI interference*^5^: I: 5.3; C: 5.0  *PCL-5*^6^: I: 12.5; C: 8,0 | | |

*Significant difference

**Abbreviations:** BPI: Brief Pain Inventory; CAGE: Cut-Annoyed-Guilty-Eye; NS: Not specified; PCL-5: PTSD Checklist for DSM-5; PROMIS: Patient-Reported Outcomes Measurement Information System; PTSD: Post Traumatic Stress disorder;

1: PROMIS scores use a T-score metric and a higher score means more of the measured characteristic. 50 is the mean of the reference population and the standard deviation is 10; 2: The pain catastrophizing score: ≥ 20 indicates high catastrophization; 3: Opioid Risk Tool was administered to identify patients at risk for opioid-related aberrant behaviors (score range, 0-26, with 0-3 indicating low risk); 4: The CAGE questionnaire is administered to identify substance abuse (score range 0-4, 2 or more is indicative of substance abuse); 5: The Brief Pain Inventory (BPI): pain severity and pain interference scores measure pain severity and interference of pain in daily activities. Several questions are scored 1-10 and the mean is used as the final measure. Higher scores mean higher levels of pain and interference; 6: The PTSD Checklist for DSM-5 (PLC-5) assesses 20 symptoms of PTSD. (score range 0-80, higher means more chances of PTSD present in patient. Scores >30 is indicative of probable PTSD)
